# Flap endonuclease 1 and DNA-PKcs synergistically participate in stabilizing replication fork to encounter replication stress in glioma cells

**DOI:** 10.1186/s13046-022-02334-0

**Published:** 2022-04-12

**Authors:** Jing Zhang, Mu Chen, Ying Pang, Meng Cheng, Bingsong Huang, Siyi Xu, Min Liu, Hao Lian, Chunlong Zhong

**Affiliations:** 1 Department of Neurosurgery, Shanghai East Hospital, Tongji University School of Medicine, 150 Jimo Road, Shanghai, 200120 China; 2 Institute for Advanced Study, Tongji University, 1239 Siping Road, Shanghai, 200092 China

**Keywords:** Glioma, DNA replication, Tumor genetic evolution, DNA damage, Genome instability, Synthetic lethality

## Abstract

**Background:**

Selectively utilizing alternative mechanisms to repair damaged DNA in essential factors deficient cancer facilitates tumor genetic evolution and contributes to treatment resistance. Synthetic lethality strategies provide a novel scenario to anticancer therapy with DNA repair protein mutation, such as glioma with DNA-PKcs-deficiency, a core factor crucial for non-homologous end joining (NHEJ) mediated DNA damage repair. Nevertheless, the clinical significance and molecular mechanisms of synthetic lethality function by interfering tumor DNA replication remain largely unexplored.

**Methods:**

Cancer clinic treatment resistance-related replication core factors were identified through bioinformatics analysis and RNA-sequencing and verified in clinical specimens by immunoblotting and in situ Proximity Ligation Analysis (PLA). Then, in vitro and in vivo experiments, including visible single molecular tracking system were performed to determine functional roles, the molecular mechanisms and clinical significance of synthetic lethality on glioma tumors.

**Results:**

Hyperactive DNA replication and regulator Flap endonuclease 1 (FEN1) provides high efficiency DNA double strand breaks (DSB) repair abilities preventing replication forks collapse during DNA replication which facilitate adaptation to selective pressures. DNA-PKcs deficient glioma cells are highly dependent on FEN1/BRCA1/RAD51 to survival and counteract replication stress. FEN1 protects perturbed forks from erroneous over-resection by MRE11 through regulating of BRCA1-RAD51 and WRN helicase, uncovering an essential genetic interaction between FEN1 and DNA-PKcs in mitigating replication-stress induced tumor genomic instability. Therapeutically, genetic depletion or molecular inhibition of FEN1 and DNA-PKcs perturb glioma progression.

**Conclusions:**

Our findings highlight an unanticipated synthetic interaction between FEN1/BRCA1/RAD51 and DNA-PKcs when dysfunction leads to incompatible with cell survival under conditions of interrupted replication progression by disrupting addictive alternative tumor evolution and demonstrate the applicability of combined FEN1 and DNA-PKcs targeting in the treatment of glioma.

**Supplementary Information:**

The online version contains supplementary material available at 10.1186/s13046-022-02334-0.

## Background

Glioma is the most prevalent primary malignant adult brain tumor and is associated with profound intra-tumoral genomic heterogeneity, which has limited therapeutic strategy development [1]. Studies based on The Cancer Genome Atlas (TCGA) and other databases have identified multiple molecular driver alterations in glioma [2, 3]. Unfortunately, targeted therapies against these abnormalities still have not achieved ideal efficacy in glioma patients [4]. These disappointing results may be due to tumor genome evolution during DNA proliferation and cell cycle progression [5, 6], which drives aggressive progression even when one factor is targeted, as cancer cells can leverage diverse complementary mechanisms to maintain genome integrity.

Effective transmission of genetic information to progeny cells is essential for maintaining genomic continuity and integrity and is dependent on multiple co-functioning proteins during DNA replication; the same conditions apply to cancer cells. However, the genome continuously suffer endogenous and exogenous replication stress driven from protein-DNA complexes, DNA secondary structures, oncogene activation, and DNA-damaging drugs such as chemotherapy medicines, causing threats to genome stability [7, 8]. Activated by single-stranded DNA (ssDNA), ATR checkpoint function is closely related to the response to the response to replication stress caused by stalled replication forks, which allows protein complexes to restore and restart forks and maintain genome integrity. Unresolved or prolonged fork stalling threatens unscheduled nucleolytic resection and leads to deleterious DNA double-strand breaks (DSBs), causing fork collapse and genome instability [9].

Forced replication fork reversal and fork remodeling have also been proposed as ways to protect stalled replication fork integrity, restore fork progression and prevent ssDNA accumulation and fork collapse under replication stress conditions [10–12]. Recent studies have revealed multiple factors that play crucial roles in the response to DNA replication stress by protecting stalled forks from degradation and promoting fork restart, including BRCA1/2, DNA-PKcs and key members of the homologous recombination (HR) and Fanconi anemia (FA) DNA repair pathways [13, 14]. Defects in DNA repair signaling systems promote genomic instability, initiating tumorigenesis [15, 16]. Enzymatically active DNA-PKcs, an important enzyme in DSB repair, is crucial for PARP-dependent recruitment of XRCC1 to stalled replication forks and effectively protects, repairs, and restarts stalled replication forks [17]. Gene mutation promotes tumorigenesis and tumor progression; however, cancer cells defective in one DNA repair pathway have evolved several overlapping and complementary DNA repair pathways, resulting in greater reliance on the alternative repair pathways for survival and proliferation, driving the DNA repair mechanism to be a “protective” in cancer cells. However, these protective mechanisms have been identified as causes of chemo-resistance, which results in challenges to clinical therapy [13]. The genomic stability of cancer cells requires the steady coordination of DNA replication fork protection, effective replication progression and cellular proliferation in response to replication stress. Genetic diversity and stress are integral to cancer evolution, and cancer cell survival is largely reliant on stress management pathways [18]. Therefore, with compromised function common in cancer cells, DNA damage repair pathways provide an Achilles heel for inducing synthetic lethality [19]. Synthetic lethality leads to inactivation of two or more genes and thus causes cell death. Synthetic lethality describes the concomitant inactivation of two genes leading to cell death in cases in which defect in either single gene is not lethal [20]. By focusing on tumor-specific genetic defects, synthetic lethality is leveraged as a therapeutic approach to promote tumor cell death while sparing normal cells [21–23].

The group of Dr. Andre Nussenzweig has made major contributions towards a detailed understanding of the primacy of the influential role of DNA repair pathway selection and related proteins on genomic stability, drug resistance/sensitivity and promotion of multiple malignancies [13, 24]. The elevated expression of the human protein FEN1 in DNA-PKcs-deficient glioma tumors affects various cellular processes and has been largely reported to be associated with cell cycle and cancer progression. As previously reported, FEN1 associates with the WRN complex to initiate efficient fork cleavage and is critical for resolving stalled replication forks [25]. Although many roles have been reported for FEN1 that are associated with DNA metabolic processes, certain functions remain enigmatic in FEN1-overexpressingglioma cells. In our previous studies, we reported the synthetic lethal interaction of miR-1193 and DNA-PKcs in glioma and the potential regulation of FEN1 signaling in mediating glioma cells survival [26]. Here, we report an unexpected role for the FEN1 protein in fork restoration that extends beyond its established function in promoting stalled fork cleavage. Our study confirms the essential role of DNA replication regulation in glioma and highlights that DNA-PKcs-deficient glioma cells are particularly addicted to complementary FEN1-mediated repair signaling. FEN1 deficiency causes unscheduled DNA strand degradation by MRE11 nuclease attack upon replication stress. Meanwhile, DNA-PKcs was also activated in other glioma cells and frequently served as a candidate for targeted therapy [27]. Therefore, we aim to explore the two combined synthetic targets for more universal therapy. In this study, we provide evidence to support a scenario in which the established FEN1 role and the DNA-PKcs interaction is crucial for DNA replication progression. The combined disruption of FEN1/DNA-PKcs interplay results in the accumulation of DSBs, increased replication fork stalling, fork collapse and genome instability, causing glioma cells lethality both in vitro and in vivo, which provides important information for developing novel strategies for clinical genotype-specific glioma-targeted therapy.

## Materials and methods

### Cell culture and transfection

M059K, M059J, U251, U87MG, LN229, T98G cells were purchased from the American Type Culture Collection. Cells free of mycoplasma contamination were maintained in Dulbecco’s modified Eagle’s medium (DMEM) or a 1:1 mixture of DMEM and Ham’s F12 medium supplemented with 2.5 mM L-glutamine, 15 mM HEPES, 0.5 mM sodium pyruvate, 1.2 g/L sodium bicarbonate, 0.05 mM nonessential amino acids and 10% fetal bovine serum. Cells were cultured at 37 °C in a humidified atmosphere containing 5% CO2. Cells were seeded in 6-well plates and transfected with indicated siRNA. SMARTpool siRNA from Dharmacon (USA) were employed to deplete the following genes: siFEN1 (L-010344–00), siWRN (L-010378–00), siMRE11 (L-009271–00), siDNA2 (L-026431–01), siBRCA1 (L-003461–00), siBRCA2 (L-003462–00), siRAD51 (L-003530–00), siRAD52 (L-011760–00), siDNA-PKcs (L-005030–00), siPARP1 (L-006656–03), siSMARCAL1 (L-013058), siZRANB3 (L-010025–01), siHLTF (L-006448–00), transfection by using Lipofectamine RNAiMAX (Invitrogen, USA) according to the manufacturer’s instructions. Cells were harvested on day 4 after transfection for further analyses.

### Antibodies and reagents

The antibodies anti-53BP1 (ab175933, 1:200 dilution), anti-RPA (ab2175, 1: 200 dilution), RAD51 (ab63801, 1:1000 dilution) and DNA2 (ab96488, 1:1000 dilution) were purchased from Abcam, USA. Antibodies anti-γH2AX (2577, 1:800 dilution), GAPDH (5174, 1:1000 dilution) were purchased from Cell Signaling, USA. BRCA1 (sc-6954) was purchased from Santa Cruz Biotechnology. Antibodies anti-SMARCAL1 (A301-616A, 1:1000 dilution), RPA2-pS4/S8 (A300-245A, 1:200 dilution), ZRANB3 (A303-033A, 1:1000 dilution) was purchased from Bethyl. BRCA2 (OP95, 1:500 dilution) was from Milipore. Antibodies specific for FEN1 (NB100-150, 1:1000 dilution), WRN (NBP1-31,895, 1:1000 dilution), HLTF (NB100-280, 1:2000 dilution) were purchased from Novus Biologicals (USA). Mouse anti-BrdU (347,580, 1:40) was purchased from BD Biosciences. AF647 (A-21247, 1:1000) and AF488 (A-11001, 1:1000) were purchased from ThermoFisher Scientific. DNA-PKcs inhibitor: NU-7441 (HY-11006) were purchased from MedChem Express (USA). MRE11 inhibitor: mirin (M9948) was purchased from Sigma-Aldrich.

### RNA sequencing

Total RNA was extracted from temozolomide (TMZ) sensitive/resistant U87MG cell lines using TRIzol reagent (Thermo Fisher Scientific, Waltham, MA, USA). RNA quality was assessed using Nanodrop2000 and Qubit 3.0. RNA integrity was determined by Agilent 2100 Bioanalyzer. Then, total RNA was treated with mRNA Capture Beads (Vazyme Biotech, Nanjing, China) to eliminate rRNAs. A VAHTS Total RNA-Seq Library Preparation Kit (Vazyme Biotech) was used for library preparation. RNA sequencing was performed on the Illumina Hiseq 2500 platform. Pair-end reads were generated with reading lengths up to 150 bp. Differentially expressed genes were identified by limma package in R (logFC > 1 and *P* < 0.05 as threshold). The heatmap was illustrated to visualize the levels of differential expressed DNA replication-related genes. The volcano plot showed the differential distribution of DNA replication and repair-related genes. The raw sequencing data have been deposited in the NCBI under BioProject accession number PRJNA768121.

### Immunofluorescence staining

M059K cells were cultured in 35 mm plates and transfected with siFEN1, sc-13, NU-7441, VX-984 or control, followed by HU treatment or not. Then cells were washed with PBS and fixed with 4% formaldehyde. Cells were permeabilized with Triton X-100 (0.05%) for 10 min, blocked with 3% BSA in PBS and then incubated overnight at 4 °C with primary antibodies. Next, cells were washed and incubated with the corresponding AF488- or AF647-conjugated secondary antibody. Finally, cells were washed with PBST for three times and stained with DAPI for 10 min at RT. Images of the mounted slides were acquired with a Zeiss Axiovert 200 M microscope.

### DNA fiber spreading analysis

DNA fiber spreading assays were performed as follows: cells were transfected with indicated siRNA or inhibitors, followed by incubation with 10 μM CldU for 20 min and then with 100 μM IdU for another 20 min. Cells were exposed to 2 mM HU for 4 h before or after IdU incubation. Cells were trypsinized and suspended in PBS, and ~ 200 cells placed on a glass microscope slide (Newcomer Glass) and 10 μl of lysis buffer (0.5% SDS in 200 mM Tris–HCl pH 7.5, 50 mM EDTA) added. DNA fibers were spread and fixed in 3:1 methanol: acetic acid, denatured with 2.5 M HCl for 1 h, neutralized in 0.4 M Tris–HCl pH 7.5 for 5 min, washed in PBS, and immunostained using anti-BrdU primary and corresponding secondary antibodies. The slides were mounted in ProLong Gold Antifade Mounting medium. Images were acquired using a Zeiss Axiovert 200 M microscope at × 63 magnification with the Axio Vision software packages (Zeiss).

### In situ Proximity Ligation Assay (PLA)

Cells were grown on 35 mm Mattek glass bottomed plates followed by indicated treatment, then cells were incubated with 0.1% formaldehyde for 5 min and then treated twice total 10 min with CSK-R buffer (10 mM PIPES, pH 7.0, 100 mM NaCl, 300 mM sucrose, 3 mM MgCl2, 0.5% Triton X-100, 300 µg/ml RNAse), and fixed in 4% formaldehyde in PBS (W/V) for 10 min at RT, followed by incubation in pre-cold methanol for 20 min at − 20 °C. After washing with PBS for three times, cells were treated with 100 ug/ml RNase in 5 mM EDTA buffer for 30 min at 37 °C. In situ PLA was performed using the Duolink PLA kit (Sigma-Aldrich) according to the manufacturer’s instructions. Briefly, the cells were blocked for 30 min at 37 °C and incubated with the respective primary antibodies for 30 min at 37 °C. Following three times washing with PBST (phosphate buffered saline, 0.1% Tween), anti-mouse PLUS and anti-rabbit MINUS PLA probes were coupled to the primary antibodies for 1 h at 37 °C. After three times washing with buffer A (0.01 M Tris, 0.15 M NaCl, and 0.05% Tween-20) for 5 min, PLA probes were ligated for 30 min at 37 °C. After three times washing with buffer A, amplification using Duolink In Situ Detection Reagents (Sigma) was performed at 37 °C for 100 min. After amplification, the plates were washed for 5 min three times with wash buffer B (0.2 M Tris 0.1 M NaCl) and one time PBS. Finally, they were coated with mounting medium containing DAPI (Prolong Gold, Invitrogen).

### Cell survival assay and cell viability assay

Cell survival fraction was assessed by evaluating the colony-forming ability. In brief, M059K, M059J and U251 cells were seeded in six-well plates (500 cells per well) after transfection with siFEN1, siNC or treatment with sc-13 or NU-7441 and were subsequently incubated for two weeks to allow colonies to develop. Cells were continuously exposed to sc-13 and NU-7441 for 14 days since the day of first treatment. The medium was replaced every 72 h with medium containing fresh FEN1 and DNA-PKcs inhibitors. Cells were finally fixed with cold methanol, and the colonies were stained with crystal violet (in a 100% methanol solution) for manual counting.

The viability of M059K, M059J and U251 cells was assessed with a Cell Counting Kit-8 (CCK8) kit (Cat#NN517, DOJINDO Laboratories, Japan) according to the manufacturer’s instructions. Cells were seeded in 96-well plates and cultured in a 37 °C incubator for up to four days after treatment, and the OD at 450 nm was measured. All cell-based assays were performed in at least triplicate.

### Migration and invasion assays

Transwell assays with or without Matrigel (Corning) were used for migration and invasion assays according to the published methods. Cells were trypsinized to single cells in Trypsin–EDTA solution after indicated treatment. Then the cells were suspended in serum-free medium and 10^5^ cells were added to the upper chamber and complete medium was added to the lower chamber for invasion assay, or migration assay without matrigel membrane. After 24 h incubation at 37 ℃, the medium was removed and the upper side of the filter was wiped, then the migrated cells on the bottom side of the filter were fixed with 4% formaldehyde and stained with crystal violet. The number of cells invading or migrating through the matrigel was counted using three randomly selected visual fields. The scale bar is 50 µm.

### Comet assay

DNA damages were detected by alkaline comet assay according to the manufacturer’s instructions (KeyGen Biotech). Briefly, cells were treated with sc-13 or NU-7441 for two days, then harvested for the followed comet assay. The cells were mixed with low melting point agarose at 37 and placed on the top layer of 0.5% normal melting point (NMP) agarose on the slide, then covered with a coverslip and placed at 4 for 5–8 min. The coverslip was gently removed and some NMP agarose was added. The slide was then covered again with a coverslip and placed at 4 for 5–8 min. Then slides were placed in chilled lysis buffer (100 mM EDTA, 2.5 M sodium chloride, 10 mM Trizma base and 1% N-lauroylsarcosinate, 1% Triton X-100) and unwinding buffer (1 mM EDTA and 300 mM sodium hydroxide), respectively, and subjected to electrophoresis. Thereafter, the slides were gently washed with 0.4 M Tris buffer, stained with GelRed DNA dye (410,003, Biotium), and visualized and analyzed under a fluorescence microscope (Zeiss). Tail moment were used to evaluate the degree of DNA damage.

### Assessment of metaphase spread and nuclear morphology

The chromosome breakage assay was performed as described previously [28]. In brief, M059K cells were treated with sc-13 or NU-7441. After four days of culture, cells were treated with 0.5% colchicine for 4 h to induce metaphase arrest, and were incubated with hypotonic solution (0.56% KCl) at room temperature for 30 min and then in a 37 °C water bath for 5 min. Fixation with pre-cooled fixation buffer (ethanol: methanol = 1:3) was repeated three times, and a dropper was used to place cells onto a clean slide. Spread cells were incubated at 55 °C overnight and stained with Giemsa solution (GS-500, Sigma) for image acquisition of aberrant chromosomes with a Zeiss Axiovert 200 M microscope.

### EdU FACS analysis

The S-phase analysis was carried out by flow using EdU staining assay according to the manufacturer’s instructions. In brief, 2 × 10^5^ M059K cells per well were seeded into 6-well plates and allowed to attach overnight. Cells were then treated with sc-13 or NU-7441, fixed with 4% formaldehyde in PBS, washed with 1% BSA and incubated for 30 min with Click-iT EdU reaction solution. After incubation, samples were washed, resuspended in 1% BSA, and analyzed with a BD FACS Calibur device and analyzed with FCS express V3 (BD Biosciences, USA).

### Xenograft tumor growth assay

In vivo efficacy studies were performed by administering 1 × 10^6^ luciferase-labeled U87MG cells (KeyGen Biotech) in the intracranial of male Nude mice. Animals were treated with either vehicle alone, sc-13 (5 mg/kg) or NU-7441 (10 mg/kg) injected intravenously through the tail vein every other day. Bioluminescence imaging was used to detect intracranial tumor growth using the IVIS Lumina LT Series III Imaging System (PerkinElmer) weekly and animals were sacrificed after five weeks inhibitor treatment for further analysis. For incidence assay, luciferase-U87MG cells were pretreated with sc-13 or NU-7441 for 48 h, followed by intracranial Xenograft tumor implanting. Tumor incidence was measured weekly by bioluminescence imaging.

### Histology and immunohistochemistry

The mice tumor samples were dissected and fixed in 10% neutral buffered formalin for at least 4 d. Then the paraffin sections of mouse tumors were deparaffinized, rehydrated, followed by processed with H&E staining. For immunohistochemistry, briefly, paraffin sections were deparaffinized and rehydrated, and blocked with 5% horse serum for 1 h at room temperature. Sections were incubated with anti-BRCA1, anti-RAD51 and anti-PARP1 antibody overnight at 4 °C. Sections were incubated with biotinylated goat anti-rat IgG (1:200, Vector Laboratories, BA9401), followed by staining with a VECTASTAIN ABC-HRP Kit (Vector Laboratories, PK-7200) according to the manufacturer’s instructions.

### TUNEL staining

The paraffin sections of mice tumors were deparaffinized, rehydrated and processed to in situ apoptosis detection using the Click-iT Plus TUNEL Assay (Life Technology, C10618) according to the manufacturer’s instructions. Images were acquired with a Zeiss Axiovert 200 M microscope.

### Western blot analysis

Cells subjected to different treatments were harvested and lysed in lysis buffer (50 mM Tris–HCl (pH 7.4), 0.15 M NaCl, and 1% Triton X-100 in PBS, supplemented with protease and phosphatase inhibitors) on ice for 30 min. Proteins were separated by SDS-PAGE on an 8–16% gel (Invitrogen) and transferred to a PVDF membrane. The membranes were blocked in 5% dry milk in 0.1% Tween-20 in PBS and detected with the indicated antibodies and the corresponding secondary antibodies. After incubation with horseradish peroxidase (HRP)-conjugated secondary antibodies (BIO-RAD), then immunoreactions were visualized using ECL western blot detection reagents (Pierce Biotechnology) and Image Lab 5.1 gel densitometry analysis system. ImageJ software (version 1.8.0.) was used to analyze protein bands. Uncropped gel images for Western blots are available in Source Data file.

### Data acquisition and analysis

We obtained the dataset of 557 glioma patients with complete clinical information and molecular data from the publicly available The Cancer Genome Atlas (TCGA) (https://www.cancer.gov/tcga). Detailed clinicopathological characteristics of glioma samples are described in Table 1. Data of 20 patients with normal brains (controls) and 325 corresponding glioma patients were acquired from CGGA(http://www.cgga.org.cn/). The “ggplot2” package was used to compare FEN1 mRNA expression between normal brains and gliomas. Kaplan–Meier curves of differential FEN1 expression were generated by the “survival” package. Images of immunohistochemistry (IHC) staining of the protein in normal brain tissues and gliomas were accessed from the Human Protein Atlas (HPA) (http://www.proteinatlas.org) [29, 30].

**Table 1:** TCGA Clinicopathological Information

	WHO II(%)	WHO III(%)	WHO IV(%)
Number			
	196	215	146
Gender			
Male	105(54%)	122(57%)	94(64%)
Female	91(46%)	93(43%)	52(36%)
Age			
	40.63 ± 0.93	45.79 ± 0.88	60.01 ± 1.093
IDH mutation			
Mutant	180(92%)	154(72%)	9(6%)
Wildtype	14(7%)	61(28%)	133(91%)
NA	2(1%)	0(0%)	4(3%)
1p19q codeletion			
Codel	76(39%)	62(29%)	0(0%)
Non-codel	120(61%)	153(71%)	141(97%)
NA	0(0%)	0(0%)	5(3%)
Median Survival Time			
	540	465	287
Censor			
Alive	174(89%)	158(73%)	52(36%)
Dead	22(11%)	57(27%)	94(64%)
NA	0(0%)	0(0%)	0(0%)

*NA* not avaliable

### Gene oncology analysis and gene set enrichment analysis

The “ClusterProfiler” package was used to explore enriched Gene Ontology (GO) items between FEN1 high and low expression phenotypes based on differentially expressed genes (DEGs)[31]. DEGs were selected by the “limma” package (|log (fold change) |> 1 and *p*-value < 0.05 as the significance threshold). Gene set enrichment analysis (GSEA) was employed to investigate the enriched pathways in FEN1 high expression glioma based on the fold changes of all genes. The significance of enrichment was evaluated by the adjusted *p*-value.

### Construction of a protein–protein interaction network

A protein–protein interaction (PPI) network was constructed using the STRING website (https://string-db.org/) and Cytoscape software [32].

### Identification of FEN1 relevant mutations

The mutation MAF files were downloaded from the TCGA and the mutation status of the FEN1 high and low expression groups were inferred via the “maftools” package [33].

### Statistical analysis

Statistical significance of PLA experiments was analyzed using the Mann–Whitney rank-sum test and expressed as mean ± SEM values. Fiber patterns and immunoblotting were analyzed using a two-sided unpaired t test, and the exact *P*-values are given in each case. These data are expressed as the mean and standard deviation (mean ± SD) values. Statistical analysis was performed using Student’s independent t-test, and two-sided *p*-values. All experiments data were calculated via GraphPad Prism 8.4.2 software to assess the significance of differences between experimental groups. For all tests: significant: *P* < 0.05, NS (not significant): *P* > 0.05. All experiments were performed at least three times, and the number of biological replicates (n) is reported in each figure legend. All bio-informatic analyses were conducted with R (version 4.1.0).

## Results

### Oncogenic role of FEN1 in glioma patients and TMZ resistant glioma cells

**Fig. 1:**
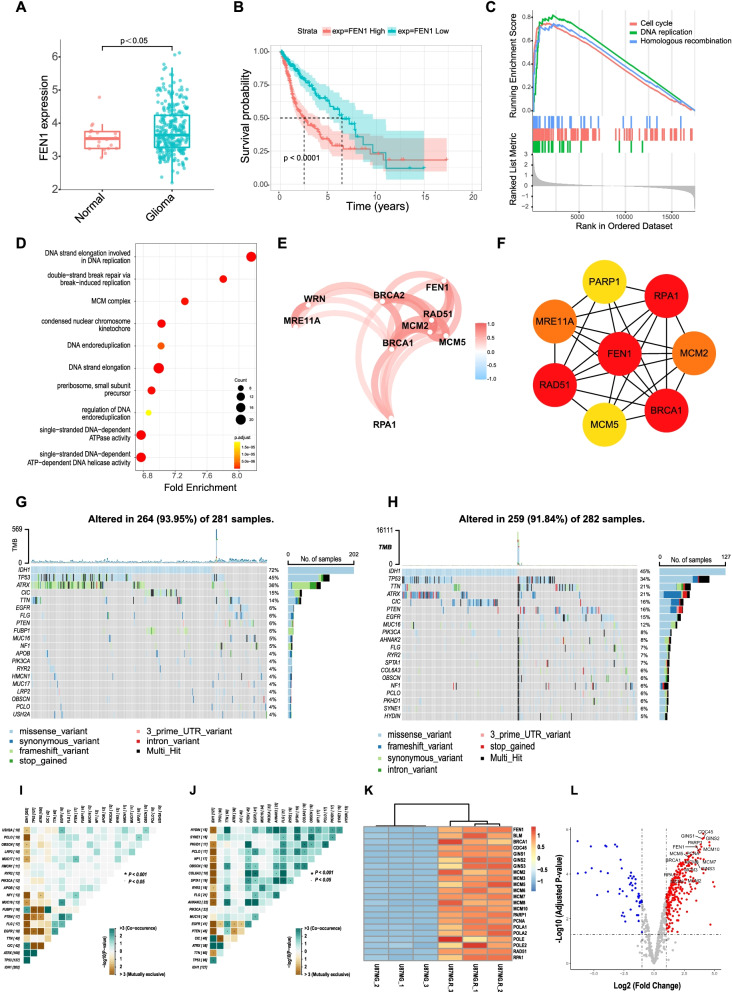
FEN1 Promotes DNA Replication Signaling Hyper-activation and Glioma Progression. **a** FEN1 expression was significantly elevated in glioma samples. **b** Overall survival of glioma patients with high and low FEN1 expression. **c** GSEA plot of DNA replication, cell cycle, and homologous recombination signatures in glioma samples. **d** GO indicates indicated a significant enrichment for multiple items of FEN1 associated with cellular progression including DNA replication and MCM complex in glioma samples **e** A correlation network plot of FEN1-related genes in glioma samples. **f** A PPI network of FEN1-related proteins. **g, h** Top 20 frequently mutated genes in FEN1 low and high expression groups. **i, j** The co-occurrence and mutually exclusive relationships between top frequently mutations in FEN1 low and high expression groups. **k** Relative mRNA level of lists of replisome factors in TMZ sensitive/resistant U87MG cells. **l** Volcano plot of DNA replication and repair-related genes in TMZ sensitive/resistant U87MG cells. Red dots indicate the up-regulated genes while blue dots indicate down-regulated genes in U87MG. R cells

To understand the expression of FEN1 in glioma cells, we used The Cancer Genome Atlas (TCGA) datasets to obtain and compare genomic data of patients with glioma and non-cancer patients. FEN1 expression was significantly overexpressed in glioma patients compared to that in non-cancer patients (Fig. 1a, Fig. S1a, Table 1). Upregulation of FEN1 expression was also found to be related to poor survival for glioma patients (Fig. 1b). A gene set enrichment analysis (GSEA) revealed significant enrichment of pathways associated with cell cycle progression, including DNA replication and the cell cycle, and homologous recombination in the glioma samples (Fig. 1c). GO analysis indicated significant enrichment for multiple pathways of FEN1 associated with cellular progression including DNA replication and MCM complex in glioma samples (Fig. 1d). These results indicated the crucial role of FEN1 in the rapid proliferation of glioma cells. The closely intertwined correlation of FEN1 and other DNA replication-related genes in glioma samples was visualized in Fig. 1e. Meanwhile, a PPI network was constructed to exhibit the interaction of FEN1-associated proteins (Fig. 1f).

To investigate the effect of high FEN1 level in glioma progression and clinical prognosis, we divided glioma samples into FEN1 high and low expression groups then investigated the FEN1-related mutational landscape. More IDH1, TP53, and ATRX mutations were observed in FEN1 low expression group, indicating a favorable prognosis that matched well with clinical diagnosis (Fig. 1g, h). Meanwhile, FEN1 high group had more PTEN and EGFR mutations and an overall higher frequency of mutation co-occurrences was uncovered, indicating an elevated mutational load (Fig. 1g-j). Increased mRNA of FEN1 and core replisome factors including MCM proteins were also observed in temozolomide (TMZ) sensitive U87MG and resistant U87MG. R cell lines by RNA sequencing (Fig. 1k). A volcano plot was graphed to show the distribution of DNA replication and repair-related genes and some significantly up-regulated members were labeled (Fig. 1l). That is, upregulated FEN1 expression results in prolonged genome stability and constitutive activation of DNA replication in TMZ resistant glioma cells.

Regarding the function of FEN1 in glioma progression, we hypothesized that a therapeutic effect can be optimized by targeting DNA replication via FEN1 inhibition and combining the effects of FEN1-mediated DNA damage signaling and clinical reagents that drive survival-related stress. In our panel of two glioma cell lines, M059K and U251 cells, we observed that FEN1 deficiency significantly and consistently reversed resistance to TMZ, cisplatin and MMS, as indicated by cell viability and survival analyses (Fig. S1b-k). Representative colony formation images indicated inhibition of the combined effects of damage reagents and FEN1 dysfunction (Fig. S1b and e). However, we did not observe significant augmentation of DNA-damage reagent cytotoxicity during FEN1 inhibition in the RPE1 non-cancer cell lines (Fig. S1f, i and k), suggesting different genetic backgrounds in the cancer and non-cancer cells and lower effects of stress-inducing agents in non-cancer somatic cells, thereby providing a promising approach to glioma clinical treatment.

### FEN1 deficiency drives excessive resection of HU-arrested DNA replication forks 

**Fig. 2:**
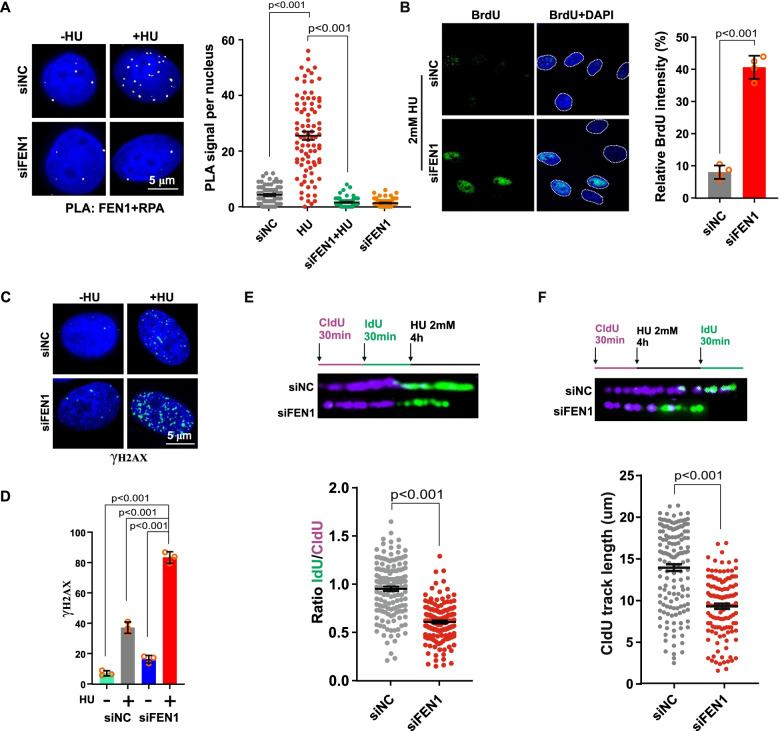
FEN1 Deficiency Inhibit Glioma Cells Proliferation, Induces Increased Replication Fork Degradation in Response to Replication Stress. **a** Detection of FEN1-RPA interaction was carried out by PLA labeling in M059K cells treated with or without 2 mM HU for 4 h. Representative images are shown. Scale bars, 5 μm. The scatterplot displays quantification of the PLA signals per nucleus from at least 100 cells from three independent experiments. Data are mean ± s.e.m. **b** M059K cells were transfected with control (siNC) or FEN1 siRNA (siFEN1) for 48 h and then treated with the indicated doses of HU for 4 h or not. Immunofluorescence labeling was performed to detect level of BrdU for ssDNA accumulation analysis. Quantitation of BrdU was presented from three independent replicates. Data are mean ± s.d. **c** Representative images of γ-H2AX by immunofluorescence labeling are shown. **d** Quantitation of γ-H2AX was presented from three independent replicates. Data are mean ± s.d. e Schematic of the CldU/IdU pulse-labeling analysis used to investigate nascent strand degradation upon HU treatment in M059K cells transfected with siFEN1 targeting FEN1 for 48 h. Representative images of CldU and IdU replication tracks (top) and scatterplot of IdU/CldU-tract length ratios (bottom) for replication forks are shown. Fiber evaluated from at least 150 events from three independent experiments. Data are mean ± s.e.m. **f** Schematic of an alternative CldU/IdU pulse-labeling protocol to investigate fork degradation upon HU treatment (2 mM, 4 h) in M059K cells transfected with siNC or siFEN1. Representative images and scatterplots of CldU tract length of individual forks are shown. Fiber evaluated from at least 150 events from three independent experiments. Data are mean ± s.e.m. For PLA experiments, a two-sided Mann–Whitney rank-sum test was used to determine if differences were significant. For immunofluorescence quantification analysis, a two-sided unpaired t test was used to calculate *P*-values. NS: not significant: *P* > 0.05

Replication stress induced by DNA-damaging reagents or deleterious structural changes during rapid cancer cell proliferation results in helicase-polymerase uncoupling, activating ATR through the accumulation of replication protein A (RPA)-coated ssDNA and increasing RPA chromatin binding, which triggers subsequent DNA damage signaling [34]. To investigate the role of FEN1 in glioma cells proliferation, we performed proximity ligation assays (PLAs) using specific antibodies against FEN1 and the heterotrimeric replication protein A (RPA) complex, which stabilizes ssDNA intermediates formed during DNA replication. Compared to that in undamaged cells, PLA signaling significantly increased in cells treated with hydroxyurea (HU), indicating that the association between FEN1 and RPA was enhanced when forks stalled under replication stress (Fig. 2a). The PLA signal was detected at a very low frequency with FEN1 was depleted following cell transfection with FEN1 siRNA. FEN1 deficiency led to increased native BrdU foci, also suggesting a role for FEN1 in inhibiting extensive replication stress-induced ssDNA accumulation (Fig. 2b). RPA facilitates FEN1 interaction with stalled replication forks and recruits downstream DNA repair factors and checkpoint kinases [25, 35]. Then, RPA2 is phosphorylated at serine 4 and serine 8 (S4/S8) by ATR, and phosphorylated RPA2 serves as a common marker for DSB repair processing and DNA replication stress. To test the impact of FEN1 deficiency on RPA2 phosphorylation at stalled replication forks induced by HU, we tested p-RPA (S4/S8) by immunofluorescence assays. We found that FEN1 depletion significantly resulted in increased RPA2 phosphorylation under HU-induced replication stress (Fig. S2a and b). γ-H2AX, a DSB maker, was also increased in cells transfected with FEN1 siRNA under HU treatment, shown by images of immunofluorescence labeling (Fig. 2c) and quantitation (Fig. 2d).

Persistent fork stalling induced by replication stress leads to excessive ssDNA accumulation, resulting in the insufficient levels of RPA available for ssDNA protection, an outcome called RPA exhaustion, which ultimately leads to single-strand DNA exposure, fork degradation and disabled fork restart in the absence of a DNA repair pathway [36]. Then, we investigated the performance of stalled forks with FEN1 deficiency in response to replication stress to gain further insights into the underlying mechanism critical for the increase in ssDNA accumulation in FEN1-depleted cells. First, we were interested in determining the role of FEN1 in protecting stalled replication forks from nucleolytic degradation, a function previously ascribed to several HR proteins, but not FEN1 [11, 14]. We carried out DNA fiber assays by labeling DNA with chlorodeoxyuridine (CldU) and iododeoxyuridine (IdU) and then exposed the cells to 4 mM HU for 4 h to cause fork stalling. FEN1 deficiency in M059K cells resulted in extensive shortening of nascent replication strands compared to control cells, indicating that stalled forks lead to more nuclease degradation (Fig. 2e). Continuous fork stalling and progressive shortening of nascent strands may be attributed to partial fork breakage due to fork degradation [37]. To further confirm that FEN1 is critical for protecting nascent strands from degradation, we induced fork stalling by treating cells with HU before performing IdU labeling a second time; this process has been described as a DNA dual-labeling scheme for inducing fork stalling by adding HU before adding the second halogenated nucleotide analog [38]. We found that the length of the first CldU-labeled replication strand was significantly shorter in M059K cells transfected with FEN1 siRNA (Fig. 2f), which was consistent with our previous findings showing that FEN1 can prevent excessive nucleolytic fork degradation. Collectively, these data indicate the important role of FEN1 in protecting stalled replication forks from degradation, which differs from its established function in the resection and repair of stalled replication forks.

### Glioma cells deficient in FEN1 are unable to cope with replication stress impaired fork progression

Studies both in vitro and in vivo indicate that FEN1 functions in a variety of DNA processes that are mediated by several important protein–protein interactions, including those of Werner syndrome protein (WRN), one of five human RecQ helicases implicated in the maintenance of genome stability and with roles in DNA replication and repair [39]. Interacting with WRN, FEN1 has also been reported to function in restarting stalled replication forks [25]. To determine the role of the FEN1-WRN complex in the DNA replication progression of glioma cells, we next measured the fraction of stalled and active replication forks in M059K cells transfected with siFEN1, siWRN and the combination of siFEN1 and siWRN. The FEN1 and WRN protein levels are shown in Fig. S2c and indicate a decline in protein expression after siFEN1 and/or siWRN transfection. We observed excessive replication fork degradation in the siFEN1- or siWRN-transfected M059K cells (Fig. S2d). Using long-term nucleotide starvation caused by HU exposure before the IdU labeling for the second time, we found that replication progression was impaired after FEN1 or WRN depletion with a significantly increased percentage of stalled forks. After cell release from HU-induced replication stress, FEN1 or WRN depletion resulted in a more than threefold increase in the number of stalled replication forks (Fig. S2e). FEN1 or WRN depletion also resulted in a decrease the number of ongoing replication forks of more than 20% after cell release from stress (Fig. S2f). FEN1 and WRN double depletion did not show additive effects on the progression of stalled forks, confirming their common function in the same fork protection pathway. Correlation analysis of 173 glioma patient samples (*r* = 0.52, *p* < 0.001) showed that FEN1 was positively correlated with WRN expression (Fig. S2g). Collectively, our data indicate that FEN1 is required for restarting replication forks stalled due to stress and for regularly maintaining cell cycle progression.

### FEN1 deficiency resulted in failure of RAD51-BRCA1 assembling and increased reversed fork degradation

**Fig. 3:**
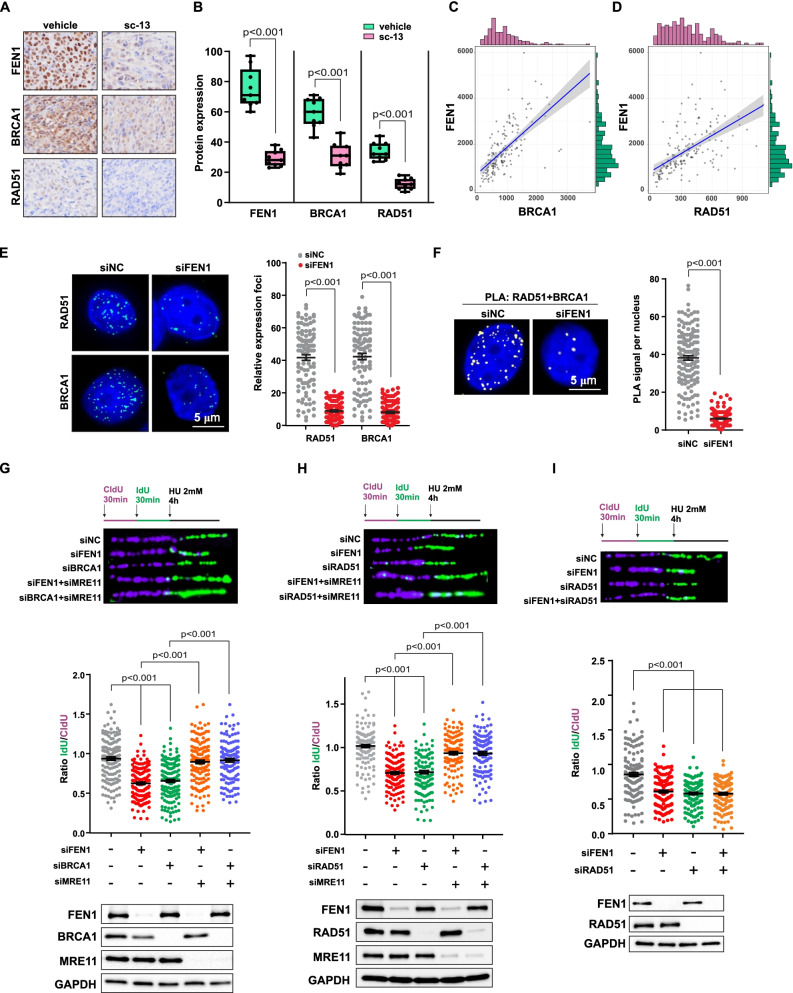
FEN1 Deficiency Resulted in Failure of RAD51-BRCA1 Assemble and Increased Fork Degradation. **a** Expression of FEN1, BRCA1 and RAD51 in glioma mice sample with or without FEN1 specific inhibitor sc-13 treatment. **b** Quantitation of FEN1, BRCA1 and RAD51 level. **c,** Correlation between FEN1 and BRCA1 protein expression in TCGA database. **d** Correlation between FEN1 and RAD51 protein expression in TCGA database. **e** M059K cells were transfected with control (siNC) or FEN1 siRNA (siFEN1) for 48 h followed with 2 mM HU treatment for 4 h. Immunofluorescence labeling was performed to detect foci of BRCA1 and RAD51. Quantitation of BRCA1 and RAD51 was presented from three independent replicates. Data are mean ± s.d. **f** Detection of BRCA1-RAD51 interaction was carried out by PLA labeling in M059K cells transfected with siNC or siFEN1 followed with 2 mM HU for 4 h. Representative images are shown. Scale bars, 5 μm. The scatterplot displays quantification of the PLA signals per nucleus from at least 100 cells from three independent experiments. Data are mean ± s.e.m. **g-i** Fork degradation was evaluated upon HU treatment in M059K cells transfected with the indicated siRNAs for 48 h. Representative images of CldU and IdU replication tracks and scatterplots of IdU/CldU-tract length ratios for individual replication forks are shown. Fibers evaluated from more than 150 counts from three independent experiments. Data are mean ± s.e.m. A two-sided Mann–Whitney rank-sum test was used to determine if differences were significant. For, NS: not significant: *P* > 0.05

Our and other studies have reported that fork remodeling by replication fork reversal allows DNA synthesis to pause and resume once the block is removed, serving as a mechanism for cells to maintain genomic stability upon replication stress [10, 12]. Mechanisms of fork protection that contribute to replication fork stability also endow cancer cells with chemo-resistance [13]. The key factors in homologous recombination (HR), BRCA1 and BRCA2, are important for stabilizing reversed forks and preventing extensive nuclease resection, as regressed arms act as entry points for unsolicited MRE11 degradation in BRCA-deficient cells [40]. Then, we studied the mechanism of FEN1 in protecting stalled replication fork degradation in response to replication stress. We labeled FEN1, BRCA1 and RAD51 in glioma mouse samples and observed decreased BRCA1 and RAD51 expression upon treatment with the specific FEN1 inhibitor sc13 [41], confirming the regulatory role of FEN1 on BRCA1 and RAD51 in glioma cells (Fig. 3a and b). Consistent with these results, there was a significant positive correlation between FEN1 and BRCA1 expression (*r* = 0.65, *p* < 0.001) and RAD51 expression (*r* = 0.56, *p* < 0.001) in the TCGA data (Fig. 3c and d). GSEA revealed significant enrichment of pathways including DNA replication, cell cycle, and homologous recombination with BRCA1 and RAD51 in the glioma samples (Fig. S3a and b). Elevated BRCA1 and RAD51 level were also observed in glioma patient samples compared with normal samples (Fig. S3c). As FEN1 depletion has been reported to impair damaged replication fork repair processing by reducing BRCA1/RAD51 function, we examined foci formation and found significantly decreased BRCA1 and RAD51 foci formation in M059K cells transfected with FEN1 siRNA compared to un-transfected cells, indicating decreased BRCA1/RAD51 recruitment to stalled forks (Fig. 3e) and markedly inhibited BRCA1-RAD51 assembly following HU treatment and observed PLA protein–protein in situ interactions in FEN1-deficient cells (Fig. 3f). Therefore, from these data, we derived a BRCA1 and RAD51 co-expression signature that demonstrated high concordance with the FEN1 signature in terms of the involved pathways and regulation by FEN1 inhibition. Therefore, FEN1 is a key regulator of BRCA1 and RAD51 protein levels and function in glioma cells (Fig. 3a-f).

We also monitored DNA strand degradation with a DNA fiber assay. As shown in Fig. 3g and Fig. S3d, HU-treated FEN1- or BRCA1-deficient cells displayed substantial degradation of nascent DNA strands, while levels of replication in wild-type cells were restored and MRE11 expression was depleted, indicating that FEN1 protect stalled replication forks from excessive MRE11 resection by stabilizing BRCA1 at stalled replication forks. BRCA1 was also critical for promoting RAD51 loading upon replication stress. Similar replication fork degradation with FEN1 depletion resulted in BRCA1 deficiency, and fork breakage was also observed in cells transfected with RAD51 siRNA (Fig. 3h). In addition, RAD51 has also been reported to be required for the accumulation of reversed forks, protecting the stalled forks in a BRCA2-independent manner in response to replication blocks [42]. In contrast to a previous report suggesting that knockdown of RAD51 can fully restore fork degradation in the absence of CTIP [43], we found that cells with RAD51 depleted displayed fork degradation to the same extent as when FEN1 is knocked down, and double depletion of FEN1 and RAD51 did not trigger fork degradation, suggesting that FEN1 and RAD51 are involved the same regulatory pathway and that they show functional variations in glioma cells under certain conditions (Fig. 3i). Protein levels were determined by in cells with siRNA transfection by western blotting. Similarly, decreases in RAD51 expression have been shown to reduce glioma cells capacity for DNA repair and increased glioma cells sensitization to radiotherapy [44]. FEN1 depletion is a promising approach to glioma therapy by reducing HR-mediated DSB repair capacity.

As extended nascent DNA strand degradation and unstable reversed forks were observed in HU-treated BRCA2-deficient cells, we monitored the influence of FEN1 depletion on BRCA2 recruitment to stalled forks as well as its function on replication forks upon HU stress. The phenotype of the BRCA2-deficient cells with excessive DNA strand degradation was similar to that of BRCA1-deficient cells, while MRE11 depletion restored the full replication progression in BRCA2- or FEN1-deficient cells (Fig. S3e). We also observed decreased BRCA2 foci formation with BRCA2 depletion, similar to that observed in FEN1 deficiency (Fig. S3f). Protein levels were determined in cells with siRNA transfection by western blotting (Fig. S3g). The working model generated to study the role of FEN1-BRCA1/2-RAD51 in protecting replication forks against MRE11 degradation (Fig. S3h). These findings indicate that FEN1 functions in stalled replication fork protection in a BRCA-dependent manner following DNA replication stress induced by HU treatment.

### Fork degradation is replication fork reversal dependent in FEN1 deficient cells 

Replication fork reversal is a phenomenon of stalled replication fork remodeling that allows temporary DNA synthesis and protects stalled fork integrity upon DNA replication stress [10]. By reannealing the nascent DNA strands to form a fourth regressed arm, stalled replication forks are remodeled into a “chicken foot” structure. DNA-damaging agents, protein-DNA complexes, or nucleotide depletion induced by hydroxyurea (HU) treatment have been shown to cause replication stress resulting in ssDNA formation and threaten genome stability [7, 8]. Fork reversal has been shown to prevent ssDNA accumulation, promote template switching and error-free lesion bypass and restore replication progression [45, 46]. Thus, fork reversal is considered a protection mechanism that resolves stalled replication forks and maintains genomic stability in response to replication stress. SMARCAL1, ZRANB3, and HLTF, three members of the SNF2 family of DNA translocases, are thought to catalyze fork reversal in response to HU-induced nucleotide depletion and replication fork stalling [47–49]. The regressed arm of reversed forks has been shown to serve as an initiation point for uncontrolled MRE11-dependent degradation in BRCA-deficient cells [40, 50]. Thus, to investigate whether FEN1 elicits its fork-protection function before or after fork remodeling, we measured the IdU: CldU ratio in DNA fibers of M059K cells transfected with FEN1 siRNA in combination with either a SMARCAL1, ZRANB3, or HLTF siRNA. As shown in Fig. S4a, depletion of each individual DNA translocase abolished fork degradation in the FEN1-deficient cells. These data suggest that fork reversal is a prerequisite for triggering extensive nascent strand degradation in FEN1-deficient cells upon HU-induced replication stress.

Moreover, DNA2 was recently reported to extensively resect the regressed arms in CTIP-deficient cells [43]. Therefore, we examined whether DNA2 is critical for nascent DNA strand degradation in FEN1-deficient cells. However, in contrast to the results obtained upon depletion of MRE11expression, co-depletion of DNA2 and FEN1 expression did not attenuate fork degradation following HU treatment, indicating that depletion of DNA2 did not restore fork stability in the FEN1-deficient cells (Fig. S4b). Protein expression was detected with the indicated antibodies (Fig. S4c and d). Collectively, these data indicate that FEN1 protects reversed replication forks from MRE11-nucleolytic attack, depending on BRCA promotion of RAD51 loading and stabilization of nucleofilaments, not by DNA2 cleavage.

### Tumor evolution drives glioma cells reliant on FEN1-dependent proliferation with DNA-PKcs Deficiency

**Fig. 4:**
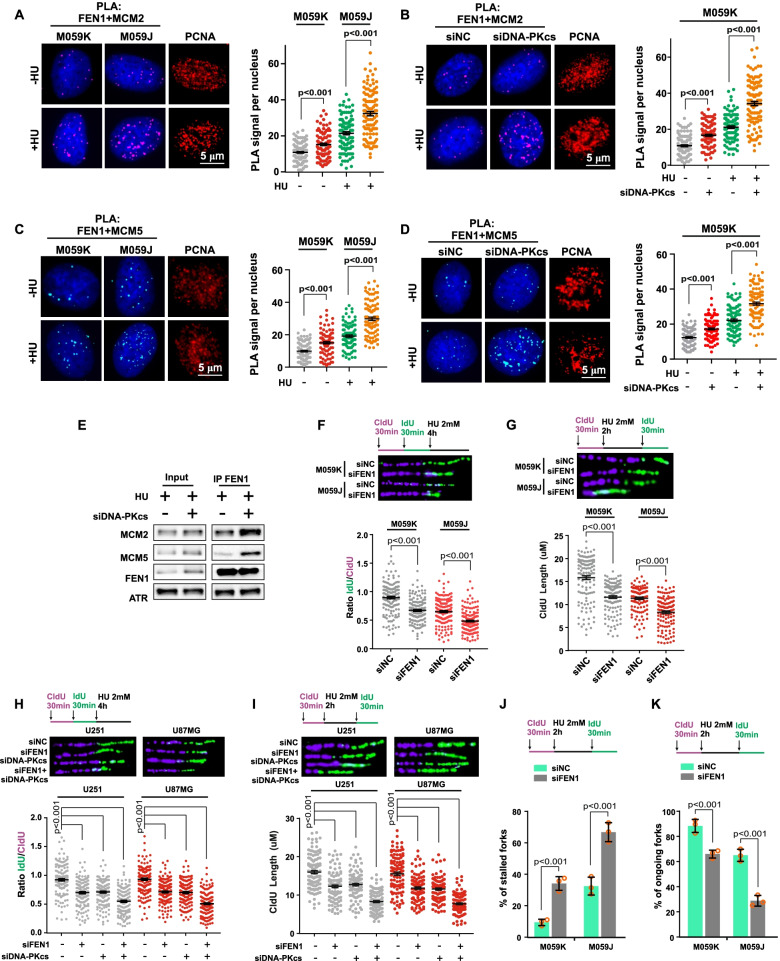
Enhanced interaction of FEN1 and Replisomes and triggered Impaired Fork Progression n with DNA-PKcs Deficiency. **a-d** Enhanced interaction of FEN1 with MCM2 and MCM5 in M059J cells or M059K transfected with siDNA-PKcs by PLA analysis. **e** Co-immunoprecipitation revealed elevated interaction of FEN1 and replisomes in DNA-PKcs deficient cells exposed to HU. **f** Fork degradation was evaluated upon HU treatment in M059K and M059J cells transfected with the siNC or siFEN1 for 48 h. Representative images of CldU and IdU replication tracks and scatterplots of IdU/CldU-tract length ratios for individual replication forks are shown. Fibers evaluated from more than 150 counts from three independent experiments. Data are mean ± s.e.m. **g** CldU length track assay with HU treatment before IdU label. Representative images of CldU and IdU replication tracks and scatterplots of IdU/CldU-tract length ratios for individual replication forks are shown. Fibers evaluated from more than 150 counts from three independent experiments. Data are mean ± s.e.m. **h, i** Fork degradation was evaluated in two additional glioma cell lines that were transfected with siFEN1, siDNA-PKcs or combined followed by HU treatment. **j, k** Schematic of an alternative CldU/IdU pulse-labeling protocol to investigate fork degradation upon HU treatment in M059K and M059J cells transfected with siNC or siFEN1. Quantification of stalled and ongoing forks are assayed. Fibers evaluated from more than 150 counts from three independent experiments. Data are mean ± s.e.m, a two-sided Mann–Whitney rank-sum test was used to determine if differences were significant **(a-d, and f-i)**. A two-sided unpaired t test was used to calculate *P*-values for stalled and ongoing forks analysis **(j, k)**. NS: not significant: *P* > 0.05

Cells utilize multiple mechanisms to maintain genome stability in response to replication stress-induced DNA damage, particularly DSB damage in cancer cells carrying the capacity for high replication progression to support their proliferation. The loss of single genes in DSB repair is not lethal because cancer cells rely on alternative DNA repair pathways. Therefore, high capacity of an effective and complementary DSB repair system contributes greatly to chemo- and radio-resistance. We next sought to determine whether FEN1 and DNA-PKcs co-depletion can block different signaling-mediated fork integrity safeguards in response to HU-induced fork stalling and DSB formation, leading to synthetic effects. Firstly, we observed significantly elevated amounts of FEN1 were recruited to replisomes as indicated by PLA analysis between FEN1 and replisomes components MCM2 and MCM5 in M059J cells (Fig. 4a and c). This finding corroborates observations in glioma cells with DNA-PKcs deficiency and in M059K cells transfected with DNA-PKcs siRNA (Fig. 4b and d) and indicates tumor evolution shifts caused by enhanced interaction between FEN1 and replisomes in cases of DNA-PKcs deficiency and FEN1 contributes to structured replisomes during cell circle. The co-immunoprecipitation assay confirmed these interactions which confirmed the enhanced interaction between FEN1 and replisomes in DNA-PKcs deficient cells (Fig. 4e). The association between FEN1 and MCM proteins was positive in control cells and increased in HU-treated cells, in agreement with the PLA assay results. These data demonstrate the shift function of FEN1 on DNA replication machine and the enhanced association of FEN1 with replisomes and alternative functions in the progression of glioma cells with DNA-PKcs dysfunction in response to replication stress.

### FEN1 deficiency triggers impaired fork progression and fork degradation in DNA-PKcs deficient glioma cells

We next carried out a DNA fiber spreading assay to examine the co-effect of FEN1 and DNA-PKcs deficiency on stalled replication forks. We observed significant fork degradation in replication-induced M059J cells upon HU treatment and triggered DNA strand resection combined upon FEN1 depletion (Fig. 4f). Then, we assayed the effect of dual depletion of FEN1 and DNA-PKcs on replication in M059K and M059J cells exposed to HU before the second DNA strand was IdU-labeled. An excessively shortened CldU-labeled strand length was observed in the M059J cells with both FEN1 and DNA-PKcs depleted (Fig. 4g). Similar phenotypes were observed in different glioma cell lines, namely, U251 and U87MG cells that were transfected with siFEN1 and siDNA-PKcs (Fig. 4h, i). FEN1 depletion resulted in an increased stalled fork frequency and a further decrease in fork progression in the M059J cells transfected with FEN1 siRNA (Fig. 4j, k).These findings indicate the synthetic effect of both FEN1 and DNA-PKcs deficiency on extensive stalled fork degradation in response to HU-induced replication stress. Protein expression significantly decreased after siFEN1 and siDNA-PKcs treatment, as determined by western blot assay (Fig. S5a-b).

To confirm this conclusion, we also analyzed fork degradation upon effective FEN1 and DNA-PKcs depletion by treating two additional glioma cell lines, U251 and U87MG cells, with the FEN1 inhibitor sc-13 [41] and the DNA-PKcs inhibitor NU-7441[51], which can be used both in vitro and in vivo. Compared to the effects on cells treated with either sc-13 or NU-7441 individually, FEN1 and DNA-PKcs dysfunction induced by treatment both inhibitors combined dramatically triggered fork degradation following HU stress induction (Fig. S5c). The same results were observed in M059J cells treated with sc-13, and fork degradation was blocked by the MRE11-specific inhibitor mirin (Fig. S5d). Triggered stalled fork frequency and a further decrease in fork progression was observed in multiple glioma cells with both FEN1 and DNA-PKcs dysfunction after specific inhibitor treatment (Fig. S5e-h). Collectively, these data imply the synthetic effect of FEN1 and DNA-PKcs depletion that leads to extensive stalled fork degradation, impaired fork restarting and additive genetic interactions in the maintenance of stalled replication fork integrity in response to HU-induced replication stress.

### Combined FEN1 and DNA-PKcs deficiency promotes replication-stress-induced DSB formation and genome instability

**Fig. 5:**
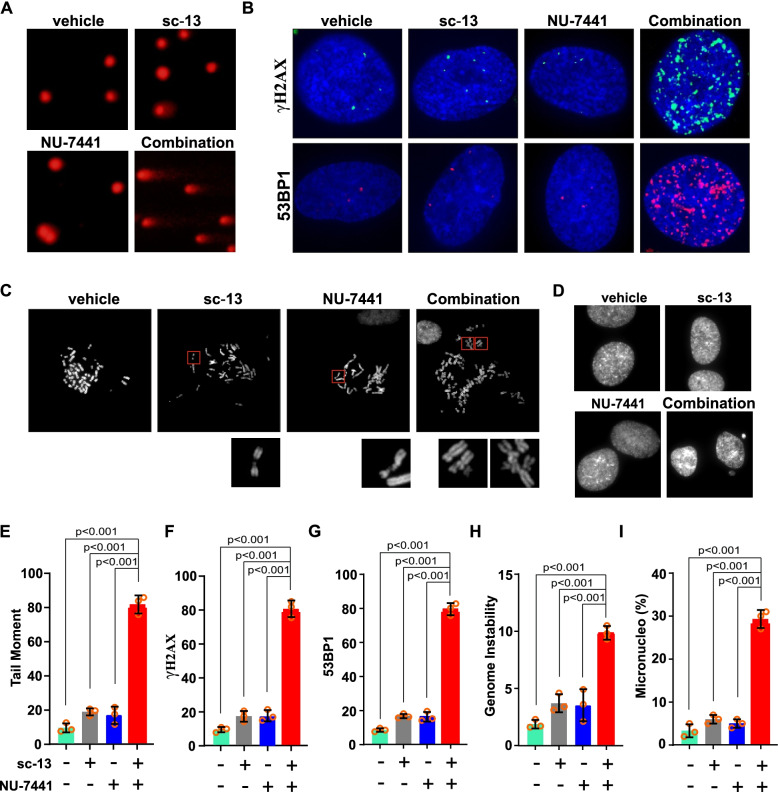
Combined Deficiency of FEN1 and DNA-PKcs Triggers DNA Damage Accumulation and Genomic Instability. **a,** Comet analysis in M059K cells treated with sc-13 or/and NU-7441 upon HU exposure. Representative images are shown. **b** Immunofluorescence labeling to detect foci of γ-H2AX and 53BP1 in cells treated with sc-13 or/and NU-7441. **c, d** Images of mitotic spreads and micronucleo forms M059K cells treated with sc-13 or/and NU-7441 following exposure to HU. Scale bar, 5 µm. e Bar chart illustrating DNA damage accumulation of comet assay. f, g Bar chart illustrating elevated level of γ-H2AX and 53BP1 in M059K cells treated with sc-13 or/and NU-7441 upon HU exposure from three independent replicates. Data are mean ± s.d. **h, i** Bar chart illustrating increased level of tail moment in M059K cells treated with sc-13 or/and NU-7441 upon HU exposure from three independent replicates. Data are mean ± s.d. A two-sided unpaired t test was used to calculate *P*-values. NS: not significant: *P* > 0.05

Prolonged replication fork stalling and failure to effectively complete replication during the S phase lead to fork collapse or breakage, followed by replication-coupled DSB damage and fork instability [52]. Therefore, we next examined the synergy between FEN1 and DNA-PKcs in response to replication stress and investigated the further functional interplay between FEN1 and DNA-PKcs on glioma cell growth. Co-inhibition of FEN1 and DNA-PKcs in M059K cells with sc-13 and NU-7441 treatment led to a significantly higher tail moment in a comet assay than that in an assay of cells treated with sc-13 or NU-7441 separately, implying attenuated DNA repair capacity of different DSB repair mechanisms in cells with deficiency in both FEN1 and DNA-PKcs (Fig. 5a and e). Moreover, γ-H2AX foci formation is widely used as a DSB damage marker, and using immunofluorescence staining, we found only mild γ-H2AX foci formation in cells with either FEN1 or DNA-PKcs deficiency. However, we observed an extended accumulation of γ-H2AX foci in FEN1/DNA-PKcs-co-deficient M059K cells (Fig. 5b and f). A similar phenotype was observed with respect to focus formation of 53BP1, another DSB marker, with results showing a synthetic effect increase in FEN1/DNA-PKcs-deficient cells (Fig. 5c and f). These findings confirmed the synthetic effect of replication fork breakage and the ultimate result of DSB accumulation upon both FEN1 and DNA-PKcs deficiency. More importantly, we observed that the combined dysfunction of FEN1 and DNA-PKcs gave rise to a striking increase in chromosomal aberrations and micronuclei formation compared to the effect of only FEN1 or DNA-PKcs depletion, suggesting extensive chromosomal instability (Fig. 5h and i). These results provide strong evidence to support the theory of the complementary interaction of FEN1 and DNA-PKcs in counteracting replication stress-induced accumulation of damaged DNA and genomic instability.

### Disruption of FEN1 and DNA-PKcs exacerbates impaired cellular progression and growth reduction

**Fig. 6:**
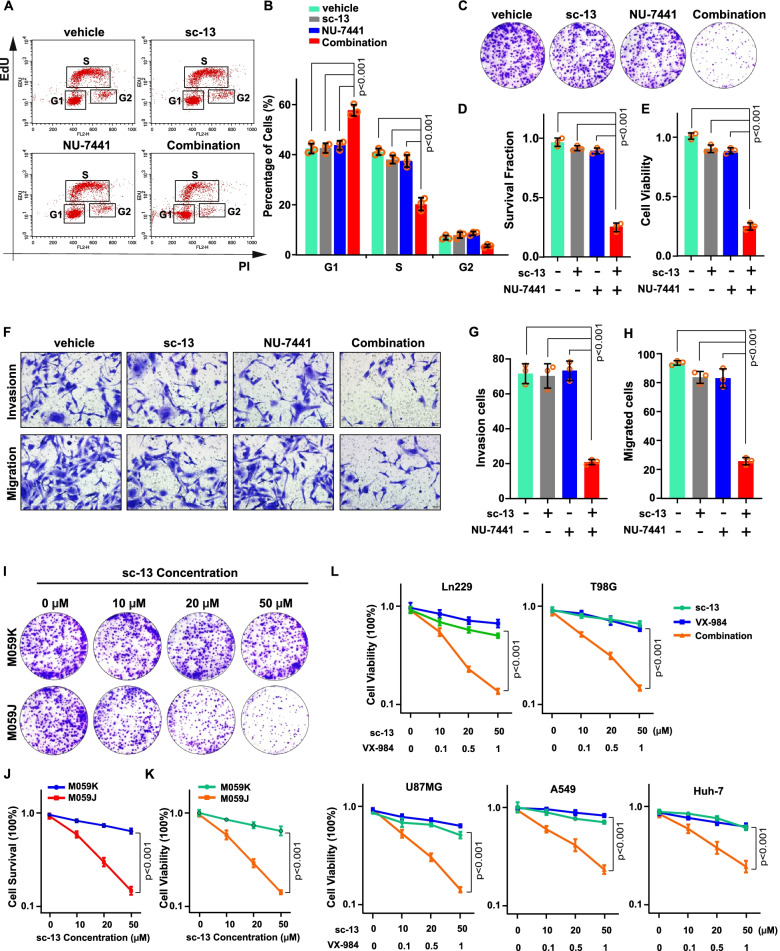
Combined Loss of FEN1 and DNA-PKcs Exacerbates Reduced Cellular Progression and Cell Viability. **a** Ethynyldeoxyuridine (EdU) detection is carried out by FACS assay. Images of different stage of cells are shown. **b** Quantification are represented as means ± s.d from at least three independent experiments. **c** Colony formation assay of U251 cells treated with sc-13 or/and NU-7441. Representative images are shown. **d** Quantification are represented as means ± s.d from at least three independent experiments. **e** Cell viability analysis are generated from CCK8 test. Data are represented as means ± s.d from at least three independent experiments. **f, g** Images and quantification of invasion and migration experiments of U251 cells treated with sc-13 or/and NU-7441 are shown. **h** Quantification are represented as means ± s.d from at least three independent experiments. **i** Clonogenic assay was performed with indicated dose of sc-13 for ten days. Representative pictures are shown. **j, k** Surviving fraction was calculated. **l** Viability assay in multiple cell lines treated with sc-13 and VX-984. A two-sided unpaired t test was used to calculate *P*-values. NS: not significant: *P* > 0.05

We next monitored the effect of FEN1/DNA-PKcs co-inhibition on glioma cells cycle progression and cell growth and observed that combined depletion of FEN1 and DNA-PKcs resulted in a reduction in EdU incorporation compared with cells depleted of either protein alone (Fig. 6a and b), suggesting that the synthetic interaction between FEN1 and DNA-PKcs counteracts DNA replication upon endogenous stress-induced impaired cell cycle progression by inhibiting DNA synthesis in the S phase. The synthetic toxicity of FEN1 and DNA-PKcs deficiency on cell growth was also observed as a reduction in the clonogenic survival fraction and short-term (four-day exposure) viability of U251 cells treated with sc-13 and NU-7441 (Fig. 6c-e). The same excessive survival inhibition was obtained in DNA-PKcs-deficient M059J cells after FEN1-specific small-molecule inhibitor treatment, compared with DNA-PKcs-proficient M059K cells (Fig. S6a-c). Strong invasion and migration are distinctive characteristics of glioma cells; therefore, we next investigated the influence of FEN1 and DNA-PKcs inhibition on the invasion and migration ability. As expected, dramatically decreased invasion and migration abilities were observed for cells with both FEN1 and DNA-PKcs depleted (Fig. 6f-h). Similar findings of excessive inhibition of invasion and migration were observed in M059J cells with combined FEN1 and DNA-PKcs depletion (Fig. S6d and e). Triggered toxicity was found in M059J cells treated with increasing concentrations of the inhibitor sc-13 (Fig. 6i-k). A similar influence of sc-13 combined with another DNA-PKcs inhibitor, vx-984, was observed in multiple glioma cells, but the extent of the effects was different in other cancer cell lines, such as A549 and Huh-7 cells, reflecting promising targeting of FEN1/DNA-PKcs in tumor therapy (Fig. 6l). Collectively, these findings consistently support a scenario in which deficiency of FEN1 and DNA-PKcs causes cell growth inhibition and aggressive decline in the migration and invasion of glioma and other tumor cells.

### In *vivo FEN1/DNA-PKcs* synthetic lethality

**Fig. 7:**
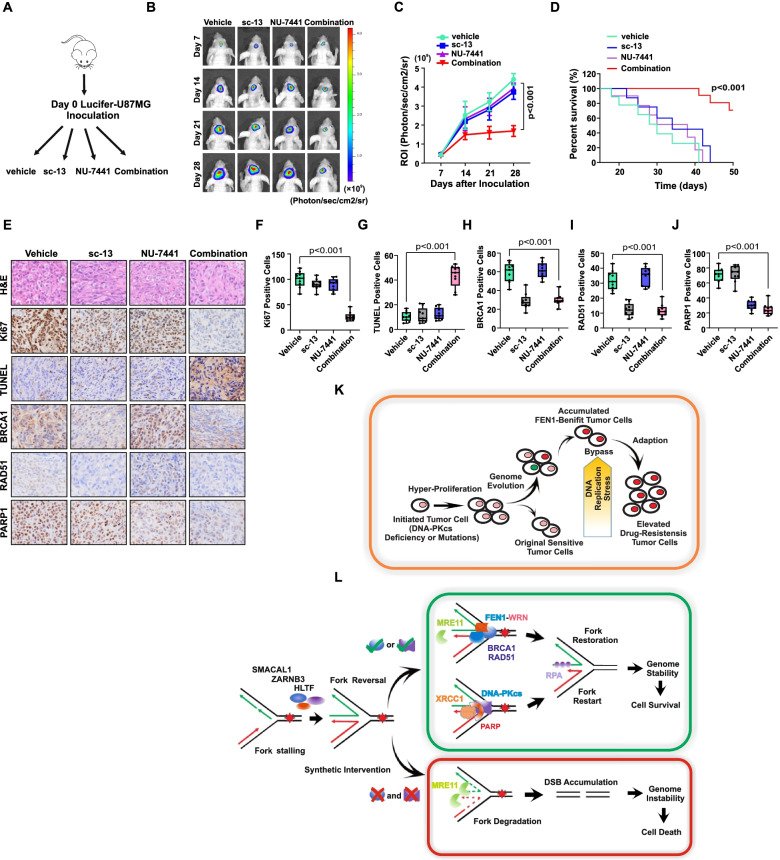
In vivo FEN1/DNA-PKcs Synthetic Lethality. **a** Schematic representation of sc-13 or/NU-7441 therapy experiment in mice bearing established Luciferase-U87 xenografts. Mice were then randomized to treatment cohorts of either sc-13 (5 mg/kg, every other day by i.v injection) or/and NU-7441 (10 mg/kg, every other day by i.v injection) or vehicle treatments. *n* = 10 mice in each cohort. Mice were treated for a subsequent 28 days. Tumor volume was detected by bioluminescence imaging weekly. **b** Representative bioluminescence imaging of each cohort at different time point. **c** ROI levels indicating reduced tumor size of sc-13 or/and NU-7441 treated mice. **d** Surviving fraction of each group was shown. **e** Tumor structure shown by H&E staining and Ki67 labeling. Expression of TUNEL, RAD51, BRCA1 and PARP1 staining determined by IHC assay in mice tumor tissue. **f-j** Quantitation of indicated protein in mice samples. **k** Model for Tumor evolution process indicating DNA-PKcs deficient glioma cells relied on FEN1-upregulated signaling pathway and survived from DNA replication stress and drug stress selection. **l** In response to replication stress, remodeling of stalled forks generate a regressed arm that form a “chicken foot” structure containing. FEN1, through stabilize and promoting BRCA1 and RAD51 assembling to the nascent strand resulting in limited nucleolytic processing of the regressed arm by MRE11. Meanwhile, by associating with WRN, FEN1-WRN complex restored the stalled forks and prevent MRE11 mediated unlimited fork degradation, ensuring stalled forks restart. DNA-PKcs, another required factor for stalled forks protection and restart, associates with PARP1 and assembled to none MRE11 resected forks promoting stalled forks degradation. Loss of FEN1 and DNA-PKcs, nascent strands at reversed forks are subjected to over-resection by MRE11, resulting in fork degradation, breakage, genomic instability and ultimately causing synthetic lethal. A two-sided unpaired t test was used to calculate *P*-values. NS: not significant: *P* > 0.05

We next assessed whether combination treatment with FEN1 and the DNA-PKcs inhibitors sc-13 and NU-7441 can inhibit glioma tumor establishment in vivo. First, we generated cohorts of null mice with xenograft tumors derived from luciferase-labeled U87MG cells. Once tumors were established, the mice were treated with either vehicle, sc-13, NU-7441 or both inhibitors for 4 weeks (Fig. 7a). The signal in the region of interest (ROI) was measured at different time points after drug treatment for up to 4 weeks. The combination of sc-13 and NU-7441 treatment significantly inhibited tumor growth compared to vehicle or sc-13 or NU-7441 treatment only. The tumor volume with sc-13 and NU-7441 treatment was dramatically lower than that of the other control groups (Fig. 7b and c). A dramatically increased surviving fraction was observed in the group of mice treated with sc-13 and NU-7441 (Fig. 7d). Sectioned brain tissue was assessed with H&E staining to determine the tumor structure accurately (Fig. 7e). As observed through immunohistochemistry labeling, significantly reduced Ki67 levels confirmed the inhibition by sc-13 and the synthetic effect of NU-7441 on glioma cells proliferation. Elevated levels of TUNEL also indicated increased tumor cell apoptosis. The levels of BRCA1 and RAD51 were significantly decreased upon sc-13 treatment, and PARP1 expression was largely reduced upon NU-7441 treatment, results in line with the findings of in vitro experiments. The superimposed deduction of BRCA1, RAD51 and PARP1 with both sc-13 and NU-7441 treatment indicated the two essential pathways critical for tumor cell replication and genome stability in response to replication stress (Fig. 7e-j).

Collectively, these in vivo experiments confirmed the synthetic lethality between FEN1 and DNA-PKcs in glioma cells.

## Discussion

A number of recent studies have suggested a DSB repair-independent role for HR factors in replication fork stabilization, thereby contributing to the maintenance of genomic integrity in multiple ways. The high expression of HR, NHEJ or other DNA repair components is related to the rapid proliferation of cancer cells, which display a hallmark of increased endogenous replication stress [8]. One DNA damage repair pathway-deficient tumor should be vulnerable to DSBs, and the reason that established mutant-gene-harboring glioma or cell lines remain viable is unclear. Tumor cells may have altered genetic or epigenetic landscapes that enable their adaptation to achieve mutant-core-gene-independent survival pathways that heavily rely on alternative DNA repair signaling. This ability of single repair factor-deficient tumor cells to adapt to endogenous or pharmacologic pressures is described in terms of tumor evolution, which leads to resistance to clinical therapies.

A previous study comparing the contribution of different DNA repair pathways to the tolerance of TMZ-O^6^MeG-induced DSB damage showed that homologous recombination plays a much more significant role than the NHEJ pathway [53]. Interestingly, FEN1 is frequently overexpressed in cancers, and its upregulation accelerates tumorigenesis in a mouse model. This outcome suggests that FEN1 is a critical component for promoting cancer cell survival and rapid proliferation during the replication stress response. As the contribution of homologous recombination is crucial for protecting tumor cells against DNA damage induced by agents such as TMZ, our finding that the HR-regulating factor FEN1 enhances DNA-PKcs-deficient glioma cell viability by playing a major role in DNA replication progression and the DNA damage response is important. The subpopulations of FEN1-upregulated cancer cells bearing selective advantages survived at the expense of the tumor evolution process and strengthens the case that homologous recombination is an important target for clinical intervention. FEN1-mediated HR replaces NHEJ in enabling the progression of stressed replication forks in DNA-PKcs-deficient cancers and is related to the malignancy of tumors and resistance to clinical treatment (Fig. 7k). FEN1 depletion has been proven to be sensitive to cisplatin treatment in lung cancer, suggesting that this novel type of anticancer agent is an effective strategy against FEN1-overexpressing cancers. Compared to chemotherapy drugs, the molecular inhibitors studied herein are expected to provide an effective and specific targeted cure for cancer with few adverse effects on normal cells.

In this study, we show that the DSB HR-mediating factor FEN1 inhibits extensive nascent strand degradation by MRE11 at arrested replication forks by stabilizing BRCA1-RAD51 loading and assembly onto stalled replication forks, contributing to HR-mediated DSB repair and promoting the progression of stalled forks, and synergistically acts with the NHEJ-mediating factor DNA-PKcs in responding to DNA replication stress. Inhibition of FEN1 induces homologous recombination deficiency through impaired expression and assembly of BRCA1 and RAD51 in multiple glioma cell types and sensitizes these cells to the effects of DNA-PKcs deficiency. The coupling of FEN1 and DNA-PKcs deficiency resulted in dramatic replication fork collapse, genome instability and synthetic lethality in glioma cells, highlighting a promising strategy for glioma therapy, particularly for glioma cells harboring oncogene mutations.

Consistent with other studies showing that even a short delay in stressed replication fork repair results in cell death, our study suggests a model by which FEN1 and DNA-PKcs synthesis maintains genome stability and aberrant combined FEN1 and DNA-PKcs processes underlie instability and synthetic lethality in glioma (Fig. 7l). In response to replication stress, remodeling of stalled forks can generate regressed arms containing a 5’ ssDNA overhang at the nascent lagging strand following effective strategies to restart replication. In regular progression, FEN1, through guaranteed BRCA1 and RAD51 assembly, ensures limited nucleolytic resection of the regressed arm by MRE11 to prevent fork degradation, ensuring fork restart. In the alternative pathway, enzymatically active DNA-PK is required for PARP-dependent recruitment of XRCC1 to stalled replication forks to establish effective protection, repair, and restart of stalled replication forks. HR-deficient cells have been shown to be highly sensitive to PARPi treatment [54], and the response to PARPi combined with BRCA1 deficiency has been shown to lead to a distinct decline in cell survival and synthetic lethality [55]. The FEN1 and DNA-PKcs proteins are individually critical for closely functioning with BRCA1 and PARP1 in the HR- and HMEJ-mediated DSB repair pathways, respectively. Thus, our findings showing that separate FEN1-BRCA1 and DNA-PKcs-PARP1 functional depletion combine to induce multiple DSB repair deficiencies resulting in replication fork collapse and genome instability also are strongly supported by these previous reports.

Moreover, as FEN1 regulates the expression and function of many molecules, there may be additional effects of FEN1 inhibition that contribute to sensitization of glioma cells to DNA-PKcs dysfunction, either cooperating with BRCA1/2, RAD51 or WRN. Thus, FEN1 inhibition-induced sensitivity to DNA-PKcs may be realized through multiple FEN1-dependent mechanisms. As DNA-PKcs-deficient glioma cells are addicted to FEN1 mediation to overcome DNA replication stress, FEN1 holds significant promise as a therapeutic agent for DNA-PKcs-deficient cancers. In the absence of FEN1 and DNA-PKcs proteins, stalled replication forks suffer impaired restart progression, followed by nascent strands at reversed forks being subjected to over-resection by MRE11, resulting in replication fork breakage and collapse, ultimately causing genomic instability and synthetic lethality. The study of tumor evolution associated with DNA replication progression has shed new insights into the neoplastic process and especially the mechanisms by which tumors harboring deficient DNA damage repair machinery escape therapy. Consistent with these results, we underscore a particularly important function in which FEN1 serves as a synthetic lethal target in DNA-PKcs-deficient glioma cells and demonstrate novel links between FEN1, BRCA1/2, RAD51, and WRN gene regulatory function and the signaling mechanism of DNA-PKcs-independent glioma cells survival and tumor growth. Importantly, the mechanism of compensatory DNA replication signaling in tumor evolution and combined DNA metabolic cytotoxicity extend and unify promising treatment strategies that may improve DNA-PKcs-mutant glioma clinical therapy.

## Conclusion

In summary, our findings provide the first evidence that FEN1 has significantly increased expression in glioma cells, tissues, and patient samples and function as an addictive dependent flat regulator for glioma cells survival. More importantly, we found an unanticipated synthetic interaction between FEN1/BRCA1/RAD51 and DNA-PKcs when dysfunction leads to incompatible with cell survival under conditions of interrupted replication progression by disrupting addictive alternative tumor evolution and demonstrate the applicability of combined FEN1 and DNA-PKcs targeting in the treatment of glioma. Further research on FEN1/DNA-PKcs may provide novel insights into glioma diagnosis and treatment, as well as significantly advance therapies in clinical personalized treatment.

## Supplementary Information

**Additional file 1. Fig. S1.** a, FEN1 expression in normal glioma patients samples. **Fig. S2.** a, M059K cells were transfected with control (siNC) or FEN1 siRNA (siFEN1) for 48 h and then treated with the indicated doses of HU for 4 h or not. **Fig. S3.** a, b, GSEA plot of DNA replication, cell cycle, and homologous recombination signatures in glioma samples. **Fig. S4.** a, b, Fork degradation was evaluated upon HU treatment in M059K cells transfected with siSMARCAL1, siZRANB3, siHLTF or siDNA2 for 48 h. **Fig S5. **a, b, Whole-cell lysates of cells were analyzed by western blotting using the indicated antibodies. **Fig S6. **a, Colony formation assay of cells transfected with siDNA-PKcs treated with sc-13.

## Data Availability

The publicly available datasets used in this study can be obtained from their respective online. All datasets used in the present study are available from the corresponding author on reasonable request.

## Abbreviations

HR: Homologous recombination
NHEJ: Non-homologous end joining
DSB: DNA double strand breaks
FEN1: Flap endonuclease 1
FA: Fanconi anemia
GSEA: Gene set enrichment analysis
RPA: Replication protein A
TCGA: The Cancer Genome Atlas
TMZ: Temozolomide
HU: Hydroxyurea
HE: Hematoxylin eosin
OS: Overall survival
KEGG: Kyoto Encyclopedia of Genes and Genomes
GO: Gene Ontology
DEG: Differentially expressed genes
PLA: Proximity Ligation Analysis
PPI: Protein–protein interaction
siRNA: Small interfering RNA
CCK-8: Cell Counting Kit-8
IHC: Immunohistochemical staining
CldU: IdU: chlorodeoxyuridine; iododeoxyuridine
EdU: 5-Ethynyl-2’-deoxyuridine
