# Supplementary material for: Flap endonuclease 1 and DNA-PKcs synergistically participate in stabilizing replication fork to encounter replication stress in glioma cells
Source: J Exp Clin Cancer Res. 2022 Apr 12;41:140. doi: 10.1186/s13046-022-02334-0 (PMC9006432; doi:10.1186/s13046-022-02334-0)
Supplement: Supplementary file 1 — Additional file 1. Fig. S1. a, FEN1 expression in normal glioma patients samples. Fig. S2. a, M059K cells were transfected with control (siNC) or FEN1 siRNA (siFEN1) for 48 h and then treated with the indicated doses of HU for 4 h or not. Fig. S3. a, b, GSEA plot of DNA replication, cell cycle, and homologous recombination signatures in glioma samples. Fig. S4. a, b, Fork degradation was evaluated upon HU treatment in M059K cells transfected with siSMARCAL1, siZRANB3, siHLTF or siDNA2 for 48 h. Fig S5. a, b, Whole-cell lysates of cells were analyzed by western blotting using the indicated antibodies. Fig S6. a, Colony formation assay of cells transfected with siDNA-PKcs treated with sc-13. [file 13046_2022_2334_MOESM1_ESM.zip › Supplementary figure legends-1.pdf]

Supplementary Figure Legends

Flap endonuclease 1 and DNA-PKcs Synergistically Participate in Stabilizing Replication Fork to Encounter Replication Stress in Glioma Cells

Jing Zhang[1,2]*, Mu Chen[1], Ying Pang[1], Meng Cheng[1], Bingsong Huang[1], Siyi Xu[1], Min Liu[1] , Hao Lian[1], Chunlong Zhong[1]*

1. Department of Neurosurgery, Shanghai East Hospital, Tongji University School of Medicine, 150 Jimo Road, Shanghai 200120, China

2. Institute for Advanced Study, Tongji University, 1239 Siping Road, Shanghai 200092, China.

Supplementary Figure Legends

Fig. S1. a, FEN1 expression in normal glioma patients samples. Representative immunohistochemistry images of FEN1 in normal brain tissues and gliomas were collected from the HPA database. b, Representative images of clonogenic assay of M059K cells. c, Survival assays of FEN1 depletion in M059K cell treated with varying concentrations of TMZ, cisplatin or MMS for ten days. d, Dose response curves of FEN1 depletion in M059K cell treated with varying concentrations of TMZ, cisplatin or MMS for four days. e, f, Representative pictures of clonogenic assays of U251 and RPE1 cells. g, Whole-cell lysates from M059K, U251 and RPE1 cells were analyzed by western blotting. h, i, Survival assays of FEN1 depletion in U251 or RPE1 cells treated with TMZ, cisplatin or MMS separately for ten days. j, k, Viability assays of FEN1 depletion in U251 or RPE1 cells treated with varying concentrations of TMZ, cisplatin or MMS for four days by CCK8 analysis. Data across panels represent mean ± s.d of three independent experiments. A two-sided unpaired t test was used to calculate P-values. NS: not significant: $P > 0.05$.

Fig S2. a, M059K cells were transfected with control (siNC) or FEN1 siRNA (siFEN1) for 48 h and then treated with the indicated doses of HU for 4 h or not. Immunofluorescence labeling was performed to detect phosphorylation of PRA on site S4/S8. b, Quantitation of p-RPA was presented from three independent replicates. Data are mean ± s.d. c, Whole-cell lysates of cells

were analyzed by western blotting using the indicated antibodies against FEN1 and WRN. d, Schematic of the CldU/IdU pulse-labeling analysis used to investigate fork degradation upon HU treatment in M059K cells transfected with siFEN1, siWRN or combined siRNA targeting FEN1 and WRN for 48 h. Representative images of CldU and IdU replication tracks and scatterplot of IdU/CldU-tract length ratios for replication forks are shown. Fiber evaluated from at least 150 events from three independent experiments. Data are mean ± s.e.m. e, f, Schematic of an alternative CldU/IdU pulse-labeling protocol to investigate fork degradation upon HU treatment in M059K cells transfected with siNC, siFEN1 or/and siWRN. Quantification of stalled and ongoing forks are assayed. Fiber evaluated from at least 150 events from three independent experiments. Data are mean ± s.e.m. g, Correlation between FEN1 and WRN protein expression in TCGA database. A two-sided Mann–Whitney rank-sum test was used to determine if differences were significant. For, NS: not significant: $P > 0.05$.

Fig S3. a, b, GSEA plot of DNA replication, cell cycle, and homologous recombination signatures in glioma samples. c, BRCA1 and RAD51 expression in glioma and normal samples. Representative immunohistochemistry images of RAD51, and BRCA1 in normal brain tissues and gliomas were collected from the HPA database. d, e, Fork degradation was evaluated upon HU treatment in M059K cells transfected with siBRCA1 or siBRCA2 for 48 h. Representative images of CldU and IdU replication tracks and scatterplots of IdU/CldU-tract length and ratios for individual replication forks are shown. Fibers evaluated from more than 150 counts from three independent experiments. Data are mean ± s.e.m. f, Immunofluorescence labeling was performed to detect foci of BRCA2 in cells transfected with siNC or siFEN1 upon HU exposure. Quantitation of BRCA2 was presented from three independent replicates. Data are mean ± s.d. g, Whole-cell lysates of cells were analyzed by western blotting using the indicated antibodies. h, Model of FEN1-BRCA1/2-RAD51 signaling in preventing fork degradation. For immunofluorescence, fork stalling and fork ongoing experiments, a two-sided unpaired t test was used to calculate P-values. For fiber assay, a two-sided Mann–Whitney rank-sum test was used to determine if differences were significant. For, NS: not significant: $P > 0.05$.

Fig S4. a, b, Fork degradation was evaluated upon HU treatment in M059K cells transfected with siSMARCAL1, siZRANB3, siHLTF or siDNA2 for 48 h. Representative images of CldU and IdU replication tracks and scatterplots of IdU/CldU-tract length ratios for individual replication forks

are shown. Fibers evaluated from more than 150 counts from three independent experiments. Data are mean ± s.e.m. For immunofluorescence, fork stalling and fork ongoing experiments. c, d, Whole-cell lysates of cells were analyzed by western blotting using the indicated antibodies.

Fig S5. a, b, Whole-cell lysates of cells were analyzed by western blotting using the indicated antibodies. Fork degradation was evaluated upon HU treatment in U251 and U87MG cells treated with FEN1 inhibitor sc-13 or/and DNA-PKcs inhibitor NU-7441. Representative images of CldU and IdU replication tracks and scatterplots of IdU/CldU-tract length ratios for individual replication forks are shown. Fibers evaluated from more than 150 counts from three independent experiments. Data are mean ± s.e.m. c, d, Fork degradation was evaluated upon HU treatment in M059K and M059J cells treated with FEN1 inhibitor sc-13 or combined with MRE11 inhibitor mirin. Representative images of CldU and IdU replication tracks and scatterplots of IdU/CldU-tract length ratios for individual replication forks are shown. Fibers evaluated from more than 150 counts from three independent experiments. Data are mean ± s.e.m. e-h, Stalled and ongoing forks were assayed with sc-13 and NU-7441 exposure. A two-sided Mann–Whitney rank-sum test was used to determine if differences were significant. NS: not significant: $P > 0.05$.

Fig S6. a, Colony formation assay of cells transfected with siDNA-PKcs treated with sc-13. Representative images are shown. b, Quantification are represented as means ± s.d from at least three independent experiments. c, Cell viability analysis are generated from CCK8 test. Data are represented as means ± s.d from at least three independent experiments. d, e, Invasion and migration of M059K and M059J cells treated with sc-13 or/and NU-7441. Quantification from d are represented as means ± s.d from at least three independent experiments. A two-sided unpaired t test was used to calculate P-values. NS: not significant: $P > 0.05$.
