# Supplementary material for: Flap endonuclease 1 and DNA-PKcs synergistically participate in stabilizing replication fork to encounter replication stress in glioma cells
Source: J Exp Clin Cancer Res. 2022 Apr 12;41:140. doi: 10.1186/s13046-022-02334-0 (PMC9006432; doi:10.1186/s13046-022-02334-0)
Supplement: Supplementary file 1 — Additional file 1. Fig. S1. a, FEN1 expression in normal glioma patients samples. Fig. S2. a, M059K cells were transfected with control (siNC) or FEN1 siRNA (siFEN1) for 48 h and then treated with the indicated doses of HU for 4 h or not. Fig. S3. a, b, GSEA plot of DNA replication, cell cycle, and homologous recombination signatures in glioma samples. Fig. S4. a, b, Fork degradation was evaluated upon HU treatment in M059K cells transfected with siSMARCAL1, siZRANB3, siHLTF or siDNA2 for 48 h. Fig S5. a, b, Whole-cell lysates of cells were analyzed by western blotting using the indicated antibodies. Fig S6. a, Colony formation assay of cells transfected with siDNA-PKcs treated with sc-13. [file 13046_2022_2334_MOESM1_ESM.zip › Supplementary Materials.pdf]

Supplementary Figures

Flap endonuclease 1 and DNA-PKcs Synergistically Participate in Stabilizing Replication Fork to Encounter Replication Stress in Glioma Cells

Jing Zhang[1,2]*, Mu Chen[1], Ying Pang[1], Meng Cheng[1], Bingsong Huang[1], Siyi Xu[1], Min Liu[1] ,
Hao Lian[1] , Chunlong Zhong[1]*

1. Department of Neurosurgery, Shanghai East Hospital, Tongji University School of Medicine, 150 Jimo Road, Shanghai 200120, China
2. Institute for Advanced Study, Tongji University, 1239 Siping Road, Shanghai 200092, China.

Fig. S1

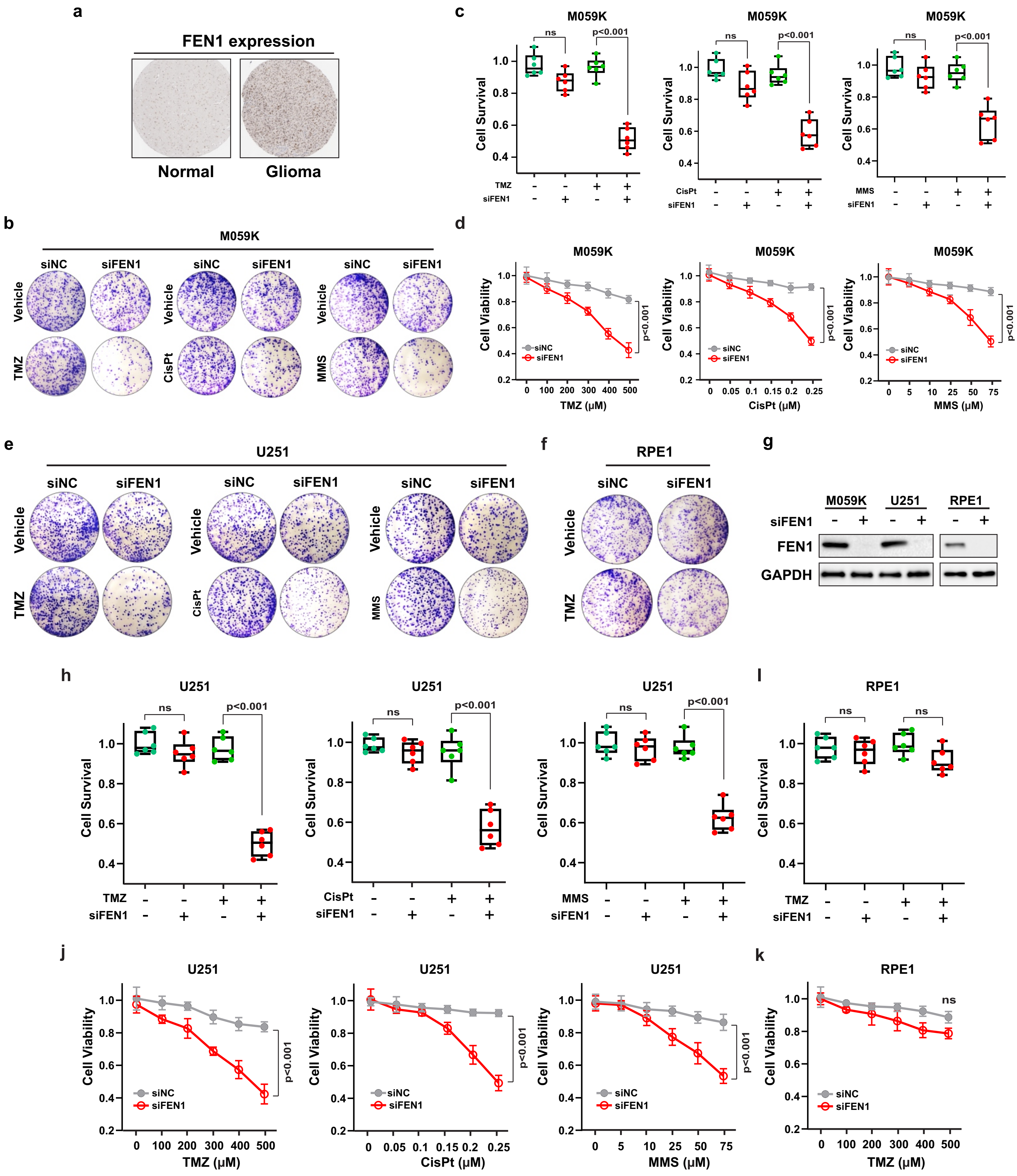

a
FEN1 expression
Normal
Glioma
b
M059K
siNC
siFEN1
Vehicle
TMZ
CisPt
MMS
c
M059K
ns
p<0.001
Cell Survival
TMZ
siFEN1
CisPt
MMS
d
Cell Viability
siNC
siFEN1
p<0.001
TMZ (μM)
CisPt (μM)
MMS (μM)
e
U251
f
RPE1
g
M059K
U251
RPE1
FEN1
GAPDH
h
i
RPE1
j
k
ns

Fig. S2

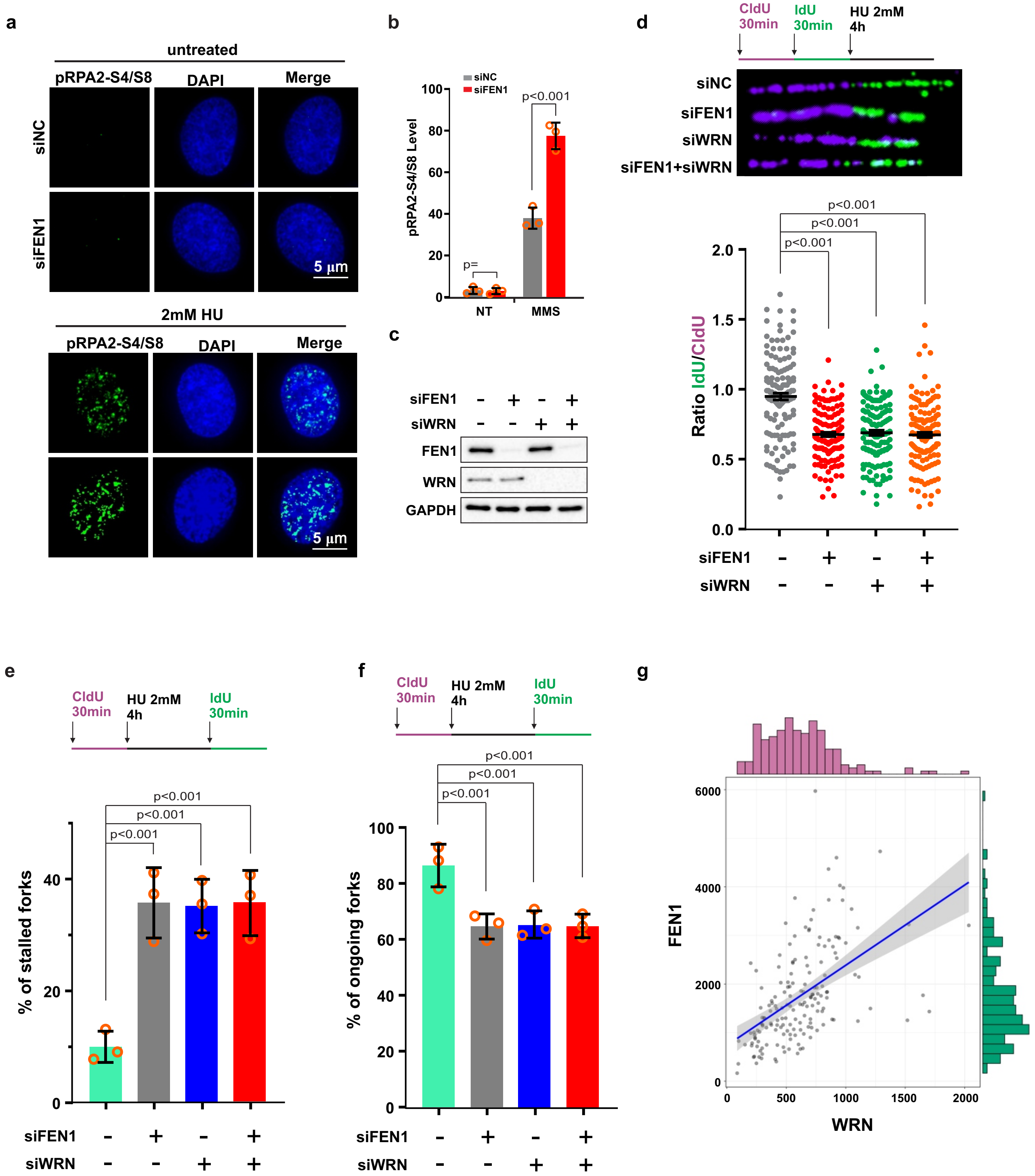
a
untreated
pRPA2-S4/S8
DAPI
Merge
siNC
siFEN1
5 µm
2mM HU
pRPA2-S4/S8
DAPI
Merge
5 µm
b
siNC
siFEN1
p<0.001
pRPA2-S4/S8 Level
p=
NT
MMS
c
siFEN1
siWRN
FEN1
WRN
GAPDH
d
CldU 30min
IdU 30min
HU 2mM 4h
siNC
siFEN1
siWRN
siFEN1+siWRN
p<0.001
p<0.001
p<0.001
Ratio IdU/CldU
siFEN1
siWRN
e
CldU 30min
HU 2mM 4h
IdU 30min
p<0.001
p<0.001
p<0.001
% of stalled forks
siFEN1
siWRN
f
CldU 30min
HU 2mM 4h
IdU 30min
p<0.001
p<0.001
p<0.001
% of ongoing forks
siFEN1
siWRN
g
FEN1
WRN

Fig. S3

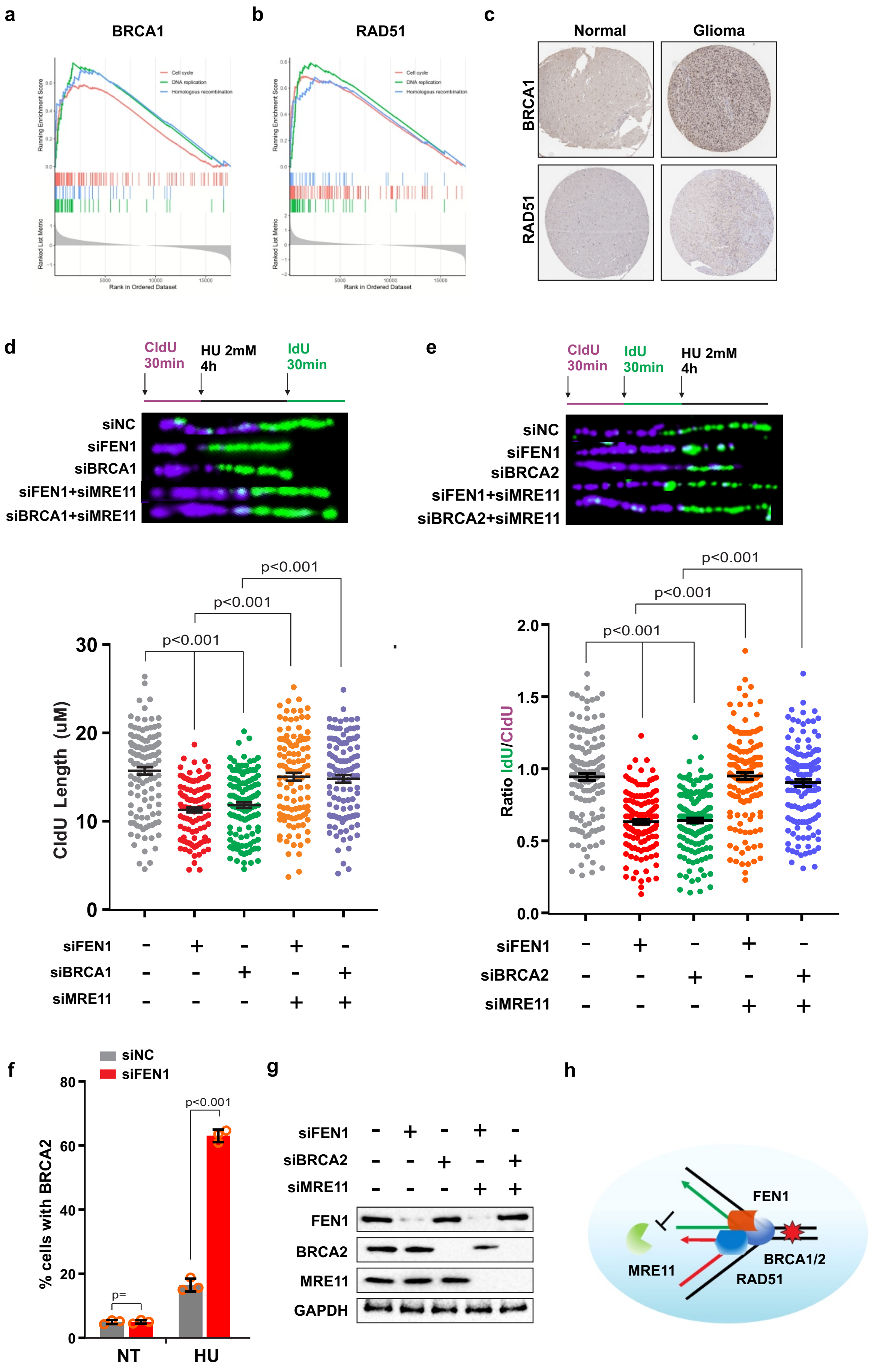
a
BRCA1
b
RAD51
c
Normal
Glioma
BRCA1
RAD51
d
CldU 30min
HU 2mM 4h
IdU 30min
siNC
siFEN1
siBRCA1
siFEN1+siMRE11
siBRCA1+siMRE11
e
CldU 30min
IdU 30min
HU 2mM 4h
siNC
siFEN1
siBRCA2
siFEN1+siMRE11
siBRCA2+siMRE11
p<0.001
p<0.001
p<0.001
CldU Length (uM)
siFEN1
siBRCA1
siMRE11
p<0.001
p<0.001
p<0.001
Ratio IdU/CldU
siFEN1
siBRCA2
siMRE11
f
siNC
siFEN1
% cells with BRCA2
p<0.001
p=
NT
HU
g
siFEN1
siBRCA2
siMRE11
FEN1
BRCA2
MRE11
GAPDH
h
FEN1
MRE11
BRCA1/2
RAD51

Fig. S4

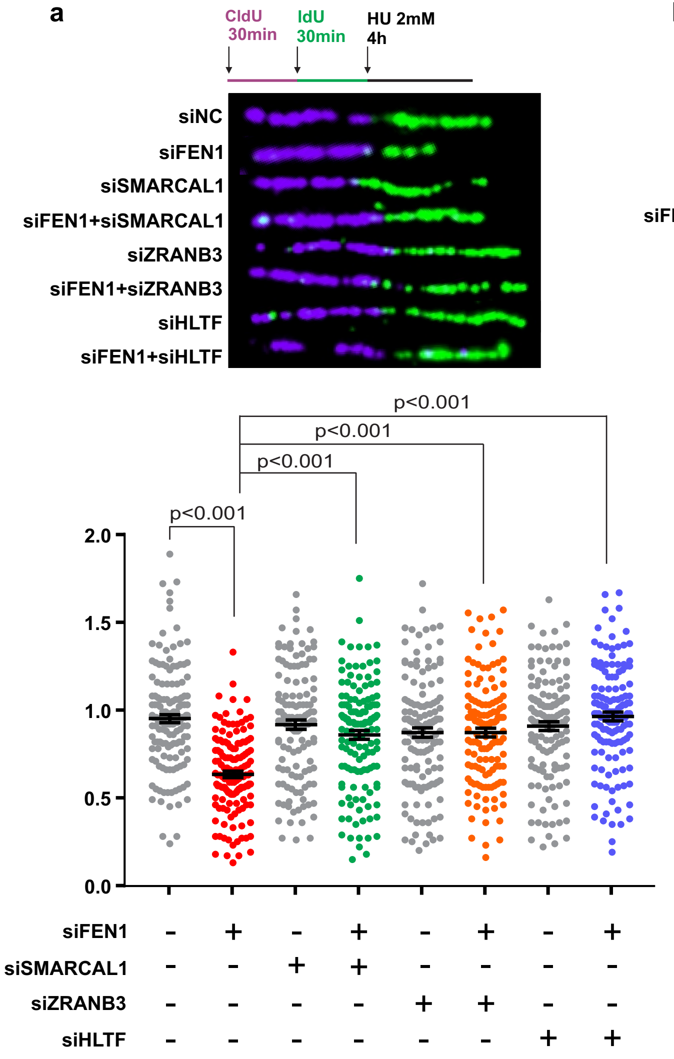
a
CldU
30min
IdU
30min
HU 2mM
4h
siNC
siFEN1
siSMARCAL1
siFEN1+siSMARCAL1
siZRANB3
siFEN1+siZRANB3
siHLTF
siFEN1+siHLTF
p<0.001
p<0.001
p<0.001
p<0.001
2.0
1.5
1.0
0.5
0.0
siFEN1 - + - + - + - +
siSMARCAL1 - - + + - - - -
siZRANB3 - - - - + + - -
siHLTF - - - - - - + +

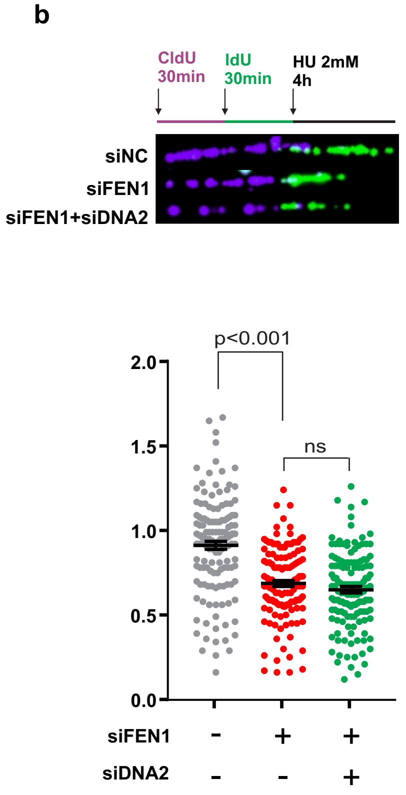
b
CldU
30min
IdU
30min
HU 2mM
4h
siNC
siFEN1
siFEN1+siDNA2
p<0.001
ns
2.0
1.5
1.0
0.5
0.0
siFEN1 - + +
siDNA2 - - +

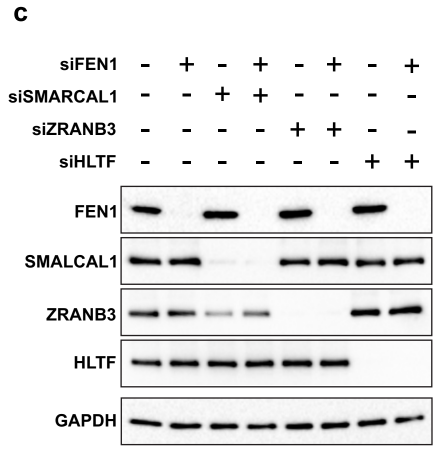
c
siFEN1 - + - + - + - +
siSMARCAL1 - - + + - - - -
siZRANB3 - - - - + + - -
siHLTF - - - - - - + +
FEN1
SMALCAL1
ZRANB3
HLTF
GAPDH

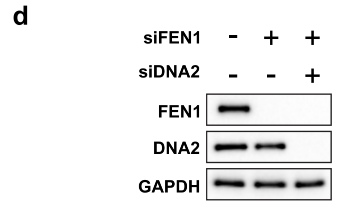
d
siFEN1 - + +
siDNA2 - - +
FEN1
DNA2
GAPDH

Fig. S5

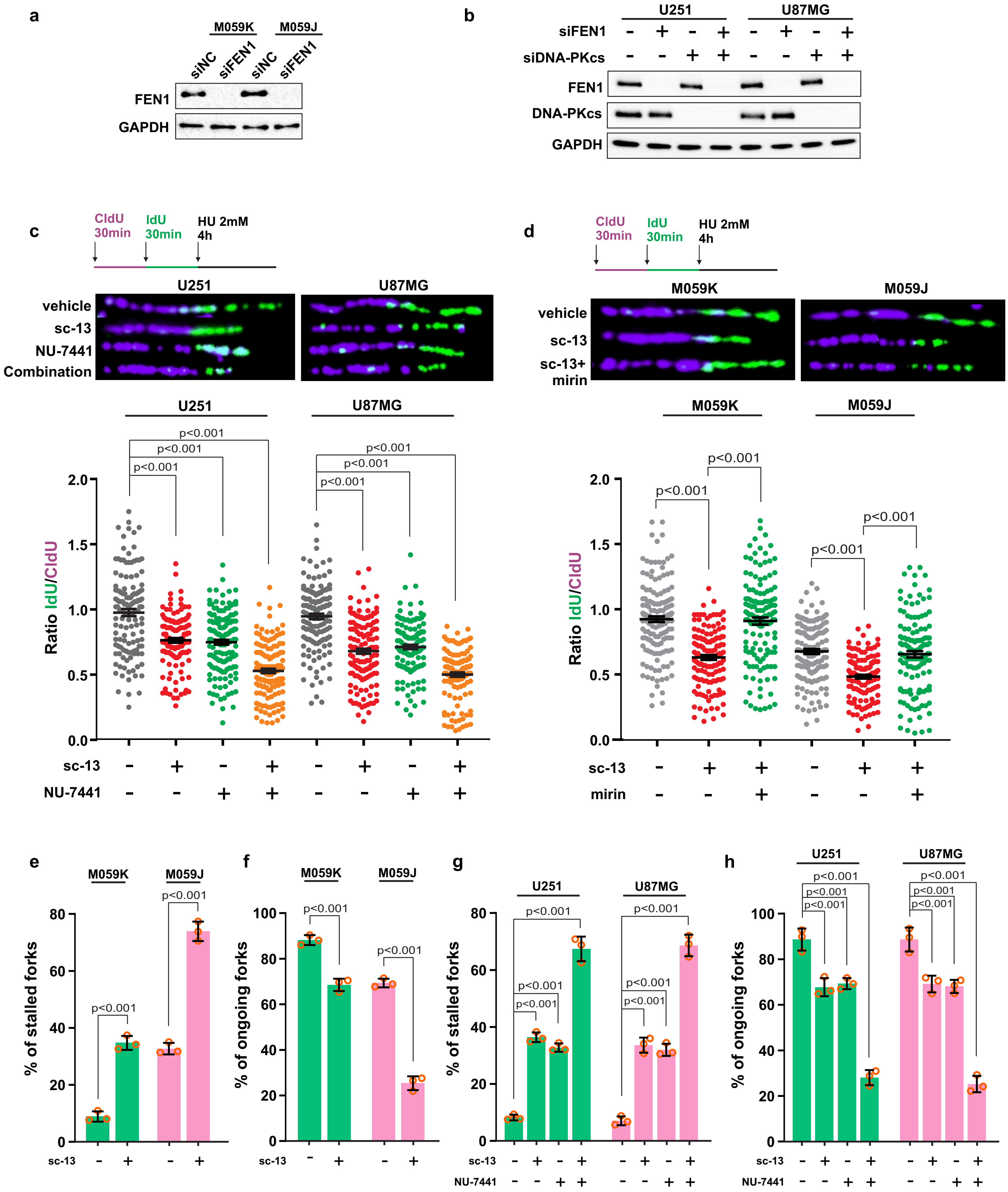

a
M059K
M059J
siNC
siFEN1
siNC
siFEN1
FEN1
GAPDH
b
U251
U87MG
siFEN1
siDNA-PKcs
FEN1
DNA-PKcs
GAPDH
c
CldU 30min
IdU 30min
HU 2mM 4h
U251
U87MG
vehicle
sc-13
NU-7441
Combination
Ratio IdU/CldU
p<0.001
sc-13
NU-7441
d
CldU 30min
IdU 30min
HU 2mM 4h
M059K
M059J
vehicle
sc-13
sc-13+ mirin
Ratio IdU/CldU
p<0.001
sc-13
mirin
e
M059K
M059J
% of stalled forks
p<0.001
sc-13
f
M059K
M059J
% of ongoing forks
p<0.001
sc-13
g
U251
U87MG
% of stalled forks
p<0.001
sc-13
NU-7441
h
U251
U87MG
% of ongoing forks
p<0.001
sc-13
NU-7441

Fig. S6

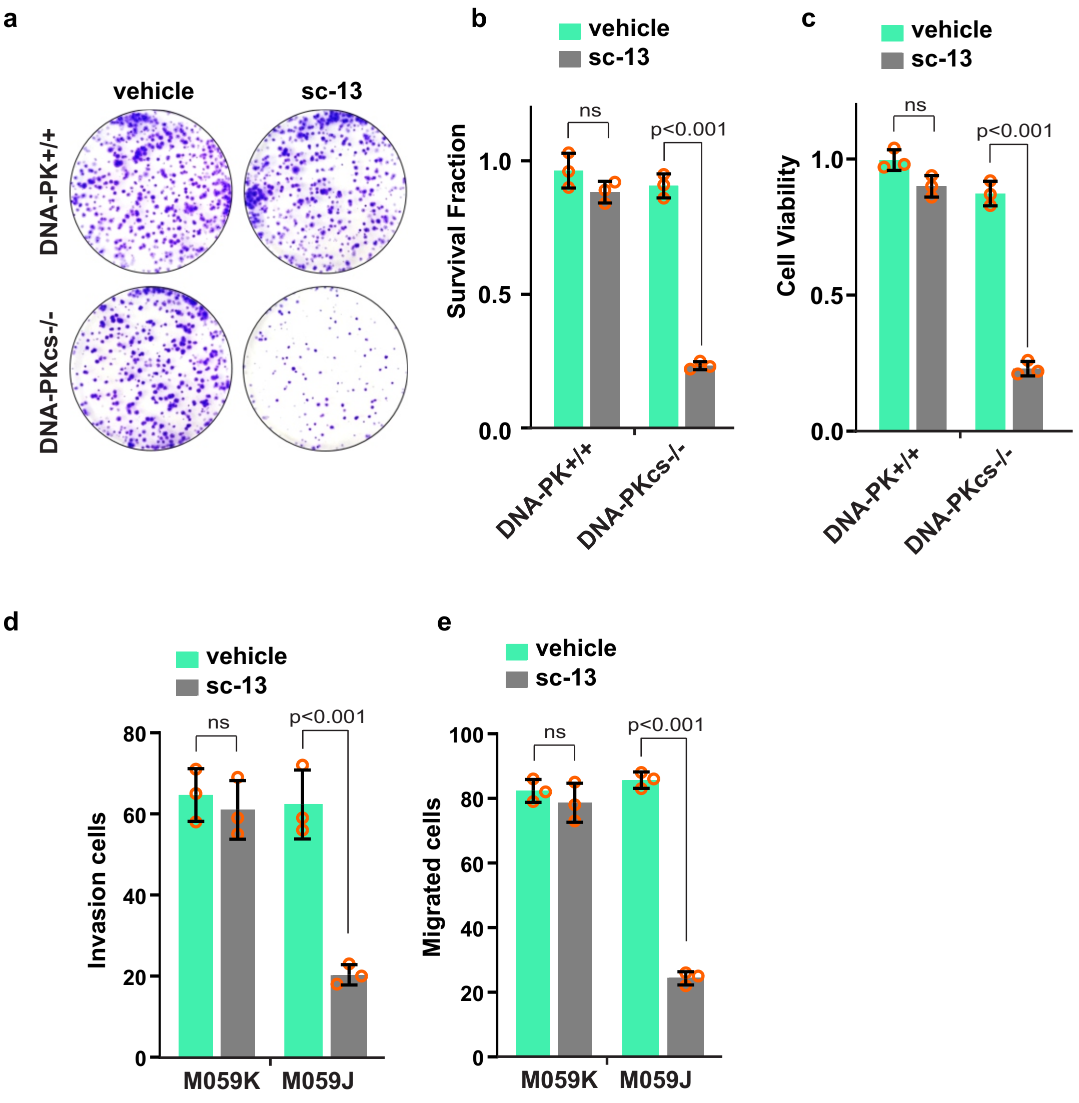

a
vehicle
sc-13
DNA-PK+/+
DNA-PKcs-/-
b
vehicle
sc-13
Survival Fraction
ns
p<0.001
1.0
0.5
0.0
DNA-PK+/+
DNA-PKcs-/-
c
vehicle
sc-13
Cell Viability
ns
p<0.001
1.0
0.5
0.0
DNA-PK+/+
DNA-PKcs-/-
d
vehicle
sc-13
Invasion cells
ns
p<0.001
80
60
40
20
0
M059K
M059J
e
vehicle
sc-13
Migrated cells
ns
p<0.001
100
80
60
40
20
0
M059K
M059J
